# T-bet^+^CD27^+^CD21^–^ B cells poised for plasma cell differentiation during antibody-mediated rejection of kidney transplants

**DOI:** 10.1172/jci.insight.148881

**Published:** 2021-06-22

**Authors:** Kevin Louis, Elodie Bailly, Camila Macedo, Louis Lau, Bala Ramaswami, Alexander Chang, Uma Chandran, Douglas Landsittel, Xinyan Gu, Geetha Chalasani, Adriana Zeevi, Parmjeet Randhawa, Harinder Singh, Carmen Lefaucheur, Diana Metes

**Affiliations:** 1 Department of Surgery, Thomas E. Starzl Transplantation Institute, University of Pittsburgh Medical Center, Pittsburgh, Pennsylvania, USA.; 2 Human Immunology and Immunopathology, INSERM UMR 976, Université de Paris, Paris, France.; 3 Center for Systems Immunology,; 4 Department of Immunology,; 5 Department of Biomedical Informatics,; 6 Department of Medicine, and; 7 Department of Pathology, School of Medicine, University of Pittsburgh, Pittsburgh, Pennsylvania, USA.

**Keywords:** Immunology, Transplantation, Chronic kidney disease, Immunoglobulins, Organ transplantation

## Abstract

Alloimmune responses driven by donor-specific antibodies (DSAs) can lead to antibody-mediated rejection (ABMR) in organ transplantation. Yet, the cellular states underlying alloreactive B cell responses and the molecular components controlling them remain unclear. Using high-dimensional profiling of B cells in a cohort of 96 kidney transplant recipients, we identified expanded numbers of CD27^+^CD21^–^ activated memory (AM) B cells that expressed the transcription factor T-bet in patients who developed DSAs and progressed to ABMR. Notably, AM cells were less frequent in DSA^+^ABMR^–^ patients and at baseline levels in DSA^–^ patients. RNA-Seq analysis of AM cells in patients undergoing ABMR revealed these cells to be poised for plasma cell differentiation and to express restricted *IGHV* sequences reflective of clonal expansion. In addition to T-bet, AM cells manifested elevated expression of interferon regulatory factor 4 and Blimp1, and upon coculture with autologous T follicular helper cells, differentiated into DSA-producing plasma cells in an IL-21–dependent manner. The frequency of AM cells was correlated with the timing and severity of ABMR manifestations. Importantly, T-bet^+^ AM cells were detected within kidney allografts along with their restricted *IGHV* sequences. This study delineates a pivotal role for AM cells in promoting humoral responses and ABMR in organ transplantation and highlights them as important therapeutic targets.

## Graphical abstract

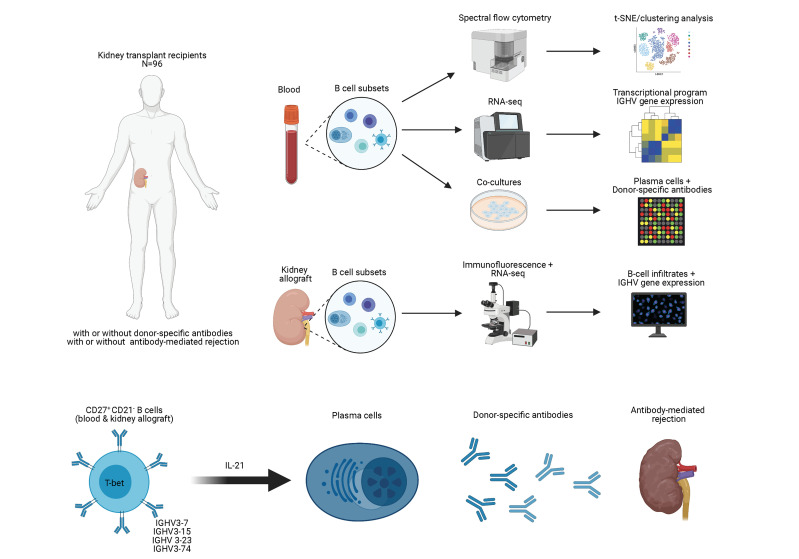

## Introduction

Humoral alloimmunity mediated by anti–human leukocyte antigen (anti-HLA) donor-specific antibodies (DSAs) significantly impedes prolonged survival of allografts after organ transplantation (1–3). Antibody-mediated rejection (ABMR) represents the complex clinical manifestation of deleterious DSA responses, which involve microvascular inflammation, arteritis, and complement activation in allograft vessels, and is associated with poor outcome (4, 5). We and others have shown that the emergence of proliferating B cells enriched for donor HLA specificity preceded the onset of ABMR, was predictive of the magnitude of DSA responses and histological damage (6–8), and was associated with increased risk of kidney allograft loss (9). The HLA-specific activated B cells were primarily contained within the memory B cell (MBC) compartment, thereby suggesting a prominent role for antigen-experienced cells in ABMR and warranting their further exploration.

Over the past decade it has become clear that specific inflammatory conditions and antigenic stimulation, particularly in the context of vaccination, autoimmunity, and chronic infections, can result in the emergence of MBCs lacking CD21 and expressing the transcription factor T-bet (10, 11). Initially described in mice, T-bet–expressing B cells, including age-associated B cells, display multiple common phenotypic and functional attributes conserved in humans, such as the overexpression of the integrin CD11c and the activation markers CD86 and CD95, as well as the downregulation of the classical B cell markers CD23, CD24, and CD38. Moreover, these cells can function as potent effectors or display features of exhaustion (12, 13). Flu or yellow fever vaccines have been shown to strongly induce CD27^+^CD21^–^ T-bet–expressing B cells that are enriched for vaccine-specific plasma cell precursors (14, 15). In lupus and rheumatoid arthritis, such CD27^+/–^CD21^–^ T-bet–expressing cells manifest an effector B cell profile with increased potential to differentiate into autoreactive plasma cells after stimulation with Toll-like receptor ligand and IL-21 signals (16–18). In contrast, during chronic malaria, HIV, and hepatitis infections, CD27^–^CD21^–^ T-bet–expressing cells dominate the B cell response but display significantly impaired function with diminished ability to secrete antibodies, suggestive of B cell exhaustion (19–23). Conversely, HIV-positive individuals mounting highly efficient IgG1^+^ and IgG3^+^ antibodies display increased frequencies of activated CD27^+^CD21^–^ T-bet–expressing cells (24), and individuals lacking these cells fail to elaborate a protective antiviral response (25). Despite these functional differences, T-bet–expressing B cells display shared features of antigen-experienced cells, such as isotype-switched IgH loci and somatic hypermutation (26).

The occurrence of T-bet–expressing MBCs and their functional as well as genomic states remain to be explored in the context of organ transplantation and ABMR. It should be noted that we and others have documented that humoral alloimmunity (e.g., DSA generation and ABMR) is dominated by skewed and exaggerated production of IFN-γ, IL-17, and IL-21 by T follicular helper cells (T_FH_) in response to donor antigens (6, 27, 28). Based on the above considerations, we undertook a multidimensional and functionally integrated characterization of the B cell responses in patients undergoing ABMR. Importantly, our cohort design enabled these B cell responses to be compared with those in transplant patients that did not undergo ABMR and either remained DSA^–^ or developed DSAs. Our overall approach, encompassing high-dimensional flow cytometry and RNA-Seq analyses of phenotypically defined B cell subsets in a cohort of 96 kidney transplant recipients, spanned unsupervised exploration of B cell states along with in-depth characterization of particular populations based on prior biological information.

Our results uncover a subset of activated memory (AM) B cells that are T-bet^+^CD27^+^CD21^–^ and whose dynamics track with both early ABMR involving preformed DSAs as well as with late ABMR associated with de novo–generated DSAs. These AM cells expressed IL-21 receptor (IL-21R), were transcriptionally poised for plasma cell differentiation, and displayed amplification of restricted *IGHV* sequences consistent with clonal expansion. Such cells differentiated into DSA-producing plasma cells when cocultured with autologous T_FH_ cells in an IL-21–dependent manner. Importantly, T-bet^+^ AM cells were detected within kidney allografts of ABMR patients along with their characteristic amplified *IGHV* sequences, supporting their pathogenic role in allograft rejection.

## Results

### Multidimensional profiling of B cell responses in kidney transplant patients.

We enrolled 96 kidney transplant recipients, who were systematically screened for circulating DSAs and allograft rejection in the first 24 months posttransplant, and identified 3 groups: patients who did not manifest DSAs or experience ABMR (DSA^–^, *n* *=* 48), those who had DSAs but did not undergo ABMR (DSA^+^ABMR^–^, *n* = 28), and patients who had DSAs and experienced ABMR (DSA^+^ABMR^+^, *n* = 20; Supplemental Figure 1A; supplemental material available online with this article; https://doi.org/10.1172/jci.insight.148881DS1). Their clinical characteristics are shown in Supplemental Table 1. Although age and sex were comparable across the groups, DSA^+^ABMR^–^ and DSA^+^ABMR^+^ patients had higher rates of retransplantation as compared with DSA^–^ patients, suggesting increased prior exposure (memory) to alloantigens (Supplemental Table 1). Among the DSA^+^ABMR^+^ patients, 12 had DSA pretransplant, and all experienced early ABMR (before 3 months). In contrast 8 DSA^+^ABMR^+^ patients did not manifest pretransplant DSAs and underwent late ABMR (after 3 months; Supplemental Figure 1B). Of the 20 DSA^+^ABMR^+^ patients, 17 displayed concomitant T cell–mediated rejection lesions (mixed ABMR), while 3 were assessed to have pure ABMR lesions.

We profiled PBMCs and sera from cross-sectional blood samples collected at the time of the following immunological events: (a) detection of posttransplant DSAs for DSA^+^ABMR^–^ patients and (b) detection of ABMR in the presence of DSAs for DSA^+^ABMR^+^ patients. For DSA^–^ patients, the blood samples were analyzed at matched time points with those from DSA^+^ABMR^–^ and DSA^+^ABMR^+^ patients (Supplemental Figure 1B). Longitudinal analyses of PBMCs and sera from representative patients were also performed (see Methods). Using multidimensional approaches, we analyzed the phenotypic, transcriptional, and functional profiles of B cells as well as their dynamics in the 3 groups of transplant recipients. Healthy control (HC) subjects served as a control group (Supplemental Table 2).

### Emergence of circulating MBCs in patients developing posttransplant DSAs and ABMR.

We used high-dimensional flow cytometry analyses of PBMCs to evaluate the frequencies of the major circulating B cell subsets (29) (transitional, naive, MBCs, and plasmablasts) among the transplant groups and HCs (Supplemental Figure 2A). We observed a significant increase in the frequencies of total B cells in DSA^+^ABMR^+^ patients as compared with DSA^–^ patients and HCs that was due to higher frequencies of MBCs and plasmablasts. Moreover, we observed a concomitant decrease in the frequencies of transitional B cells in DSA^+^ABMR^+^ patients (Supplemental Figure 2B).

### MBCs associated with ABMR are heterogeneous and include expanded T-bet–expressing subsets.

**Figure 1:**
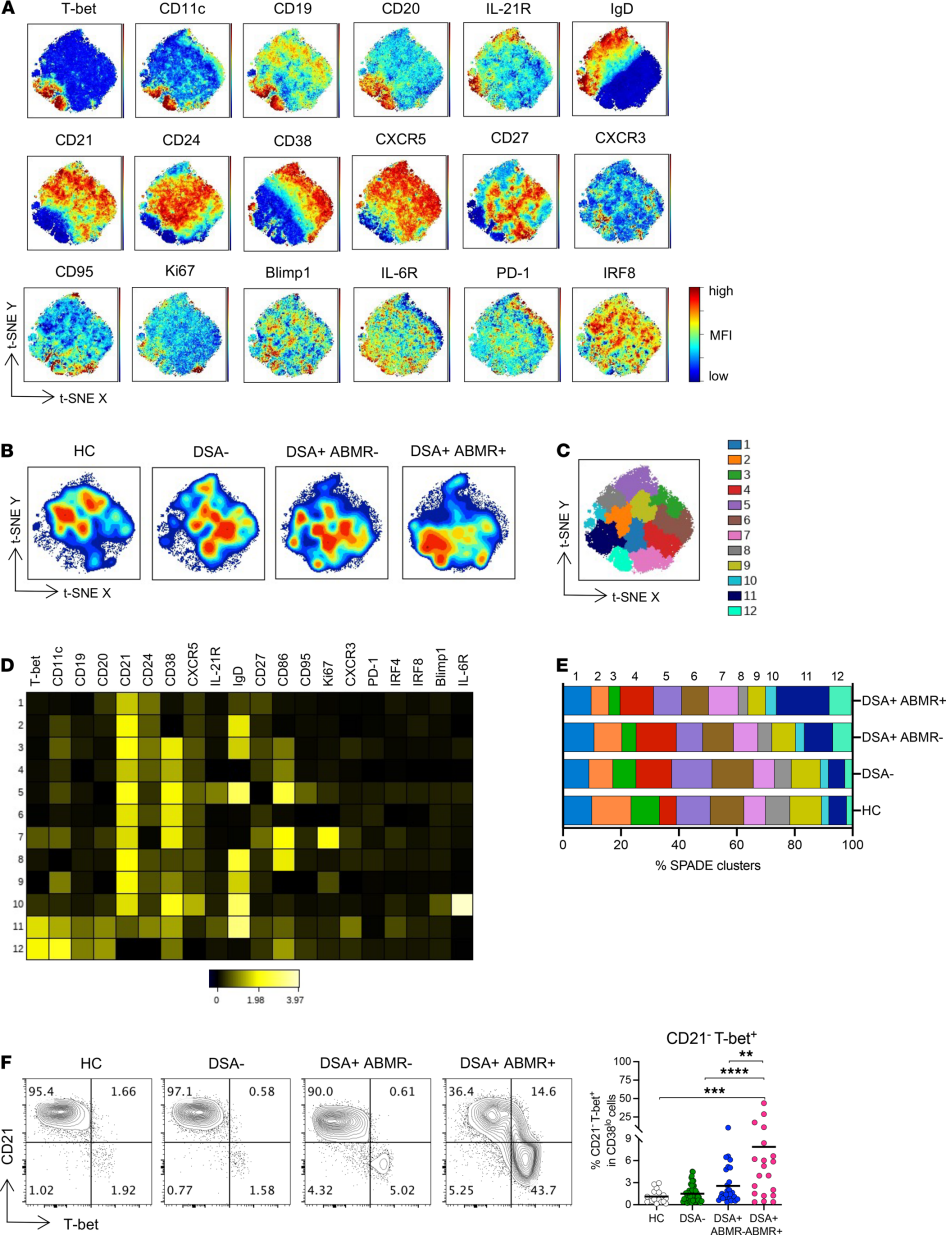
High-dimensional flow cytometry analyses of MBCs in kidney transplant patients. (**A**) t-SNE projections were generated using a concatenated file of *n* = 79,200 MBCs from HC (*n* = 4), DSA^–^ (*n* = 20), DSA^+^ABMR^–^ (*n* = 20), and DSA^+^ABMR^+^ (*n* = 20) patients; panels display expression levels of indicated markers (MFI). (**B**) t-SNE projections of MBC densities in the 4 groups using *n* = 19,800 cells from each group shown in panel **A**. (**C**) t-SNE map overlaid with 12 MBC clusters delineated by SPADE clustering of the concatenated file, as in panel **A**. (**D**) Heatmap showing the expression of markers for each MBC cluster according to transformed MFI ratio. (**E**) Stacked bar plot showing MBC cluster distribution based on SPADE clustering as in panel **C**. Clusters 3, 4, 6, 7, 9, 11, and 12 are significantly different in their proportions across the indicated groups. Kruskal-Wallis with Dunn’s posttest. (**F**) Representative examples of flow cytometry analysis and dot plot of percentages of CD21^–^T-bet^+^ cells in CD38^lo^ B cells are displayed; HC (*n* = 17), DSA^–^ (*n* = 48), DSA^+^ABMR^–^ (*n* = 28), and DSA^+^ABMR^+^ (*n* = 20) patients. Kruskal-Wallis with Dunn’s posttest. **P < 0.01; ***P < 0.001; ****P < 0.0001. Each dot represents 1 subject and horizontal lines are mean values ± SEM. SPADE, spanning-tree progression analysis of density-normalized events; t-SNE, t-distributed stochastic neighbor embedding.

To investigate the phenotypic states of MBCs, including the testing of our hypothesis pertaining to T-bet–expressing cells, we performed unbiased high-dimensional t-SNE analyses and created consensus t-SNE maps on MBCs (gated as in Supplemental Figure 2A) and based on the expression of 20 markers (Figure 1A). We observed prominent differences in MBC profiles among the various groups (Figure 1B), with a cluster of T-bet–expressing cells that had downregulated CD21, markedly distinguishing DSA^+^ABMR^+^ from other patients and HCs. T-bet^+^ cells also coexpressed CD11c, CD19, and CD20, as well as Ki67, CD95, and CXCR3. Conversely, these cells downregulated CD24, CD38, and CXCR5, and therefore, were highly distinctive from T-bet^–^ MBCs. Interestingly, T-bet^+^ cells displayed disparate expression of CD27 and IgD (Figure 1A). We next used SPADE clustering to further resolve MBC subpopulations (Figure 1C). Among the 12 SPADE clusters, high T-bet expression along with low CD21 robustly demarcated clusters 11 and 12 from all other clusters (Figure 1D and Supplemental Table 3). Cluster 11 was further distinguished from cluster 12 by higher expression of IL-21R, CXCR3, and IgD. These 2 clusters in addition to the T-bet^lo^ cluster 7 were significantly expanded in DSA^+^ABMR^+^ patients (Figure 1E and Supplemental Figure 3). The frequencies of T-bet^+^CD21^–^ cells could represent up to 40% of MBCs in these patients (Figure 1F). We note that MBCs, when compared with naive, transitional, and plasmablast compartments, exhibited the highest fractions of T-bet^+^ cells (Supplemental Figure 4). Thus, consistent with our hypothesis, DSA^+^ patients undergoing ABMR manifest increased frequencies of MBCs expressing high levels of T-bet.

### Expansion of T-bet–expressing AM and tissue-like memory cells during ABMR.

**Figure 2:**
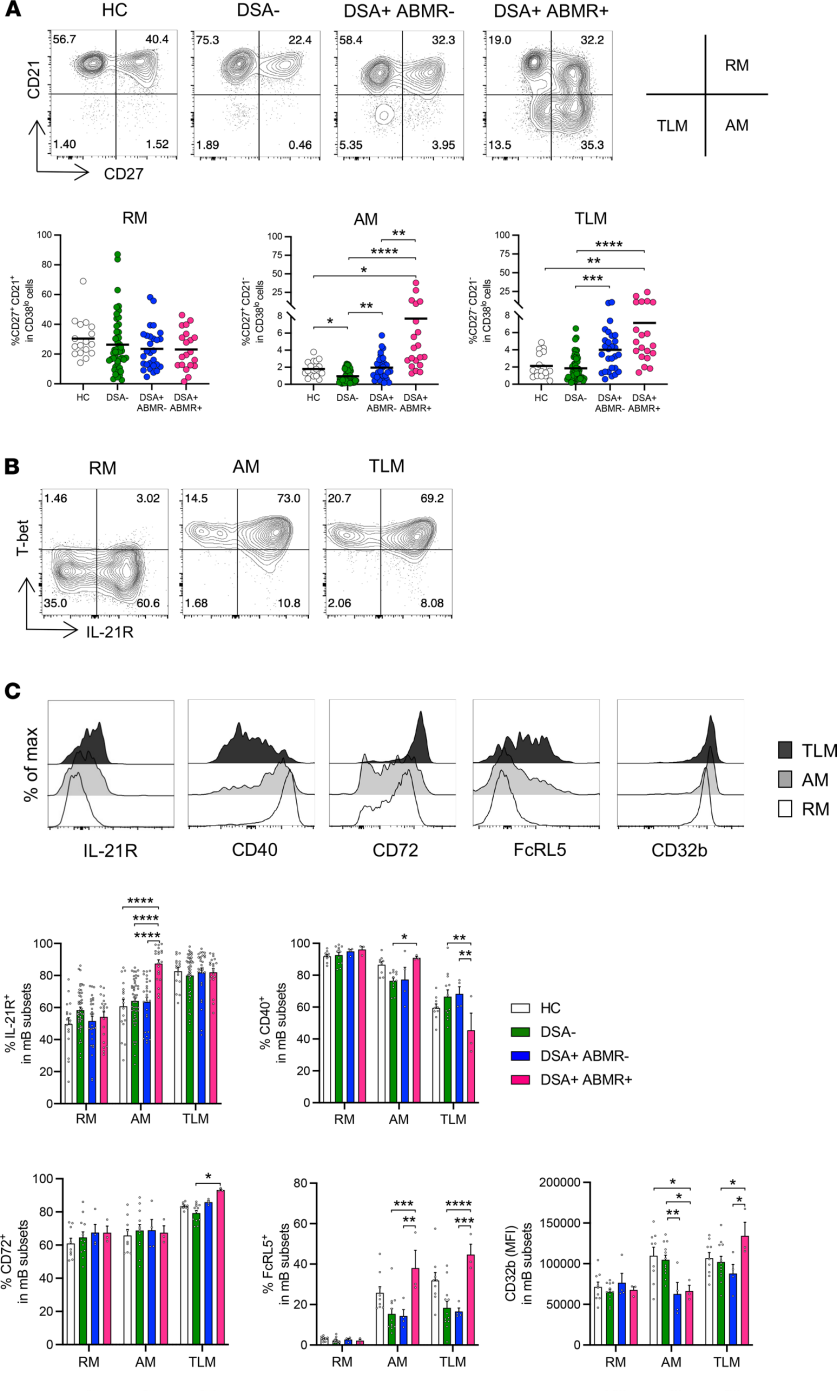
Identification of 3 distinct MBC subsets and analysis of their activation and inhibitory receptors by flow cytometry. (**A**) Representative examples of flow cytometry analysis and dot plots of percentages of resting memory (CD27^+^CD21^+^, RM), activated memory (CD27^+^CD21^–^, AM), and tissue-like memory (CD27^–^CD21^–^, TLM) subsets in CD38^lo^ B cells are displayed; HC (*n* = 17), DSA^–^ (*n* = 48), DSA^+^ABMR^–^ (*n* = 28), and DSA^+^ABMR^+^ (*n* = 20) patients. (**B**) Representative examples of flow cytometry analysis of T-bet and IL-21R in RM, AM, and TLM subsets are displayed. (**C**) Representative examples of flow cytometry histograms and bar plots of percentages of IL-21R^+^ in RM, AM, and TLM subsets are displayed; sample size as in panel **A**. Percentages of CD40^+^, CD72^+^, and FcRL5^+^ cells and MFI values of CD32b expression in RM, AM, and TLM subsets are displayed; HC (*n* = 9), DSA^–^ (*n* = 12), DSA^+^ABMR^–^ (*n* = 4), and DSA^+^ABMR^+^ (*n* = 3) patients. Kruskal-Wallis with Dunn’s posttest for panels **A** and **C**. *P < 0.05; **P < 0.01; ***P < 0.001; ****P < 0.0001. Each dot represents 1 subject and horizontal lines are mean values ± SEM.

Based on the above clustering analyses and previous studies (14, 15, 30), we used a targeted gating approach on MBCs to further segregate CD21^–^ B cells based on their expression of CD27 into CD27^+^CD21^–^ AM and CD27^–^CD21^–^ tissue-like memory (TLM) cells. Their resting memory (RM) counterparts were identified as CD27^+^CD21^+^ cells. A significant expansion in AM cell frequencies (Figure 2A) and their absolute counts (Supplemental Figure 5A) were observed in DSA^+^ABMR^+^ patients. Cellular expansion was observed at a lesser extent for TLM cells in these patients. Notably, the expression of T-bet, and that of CD11c, were restricted to AM and TLM cells and not detected in RM cells. The expression of T-bet was significantly increased in both AM and TLM cells in DSA^+^ABMR^+^ patients but was the highest in the latter subset (Supplemental Figure 5B). AM cells were more heterogeneous, consisting of SPADE clusters 4, 7, 11, and 12, as compared with TLM cells that resided in clusters 11 and 12 (Supplemental Table 3). The AM cluster 7, and the common AM/TLM cluster 11, were substantially enriched in DSA^+^ABMR^+^ patients, which distinguished them from the other patient groups (Supplemental Figure 3). We next evaluated the functional potential of AM and TLM cells by analyzing the expression of activating and inhibitory receptors. IL-21R, a critical regulator of activated B cells (31), was found to be coexpressed with T-bet (Figure 2B) and upregulated in AM cells from DSA^+^ABMR^+^ patients (Figure 2C). In addition to IL-21R, the expression of CD40, a crucial coreceptor that permits efficient response to CD40L costimulatory signals provided by T_FH_ cells, was increased in AM cells in DSA^+^ABMR^+^ patients and markedly reduced on TLM cells (Figure 2C). In contrast, TLM cells displayed increased expression of the inhibitory receptors CD72, FcRL5, and CD32b in these patients. These results suggested that AM cells, unlike TLM cells, were functionally poised to interact with cognate T_FH_ cells.

### AM cells are poised for plasma cell differentiation and enriched for distinct IGHV genes during ABMR.

**Figure 3:**
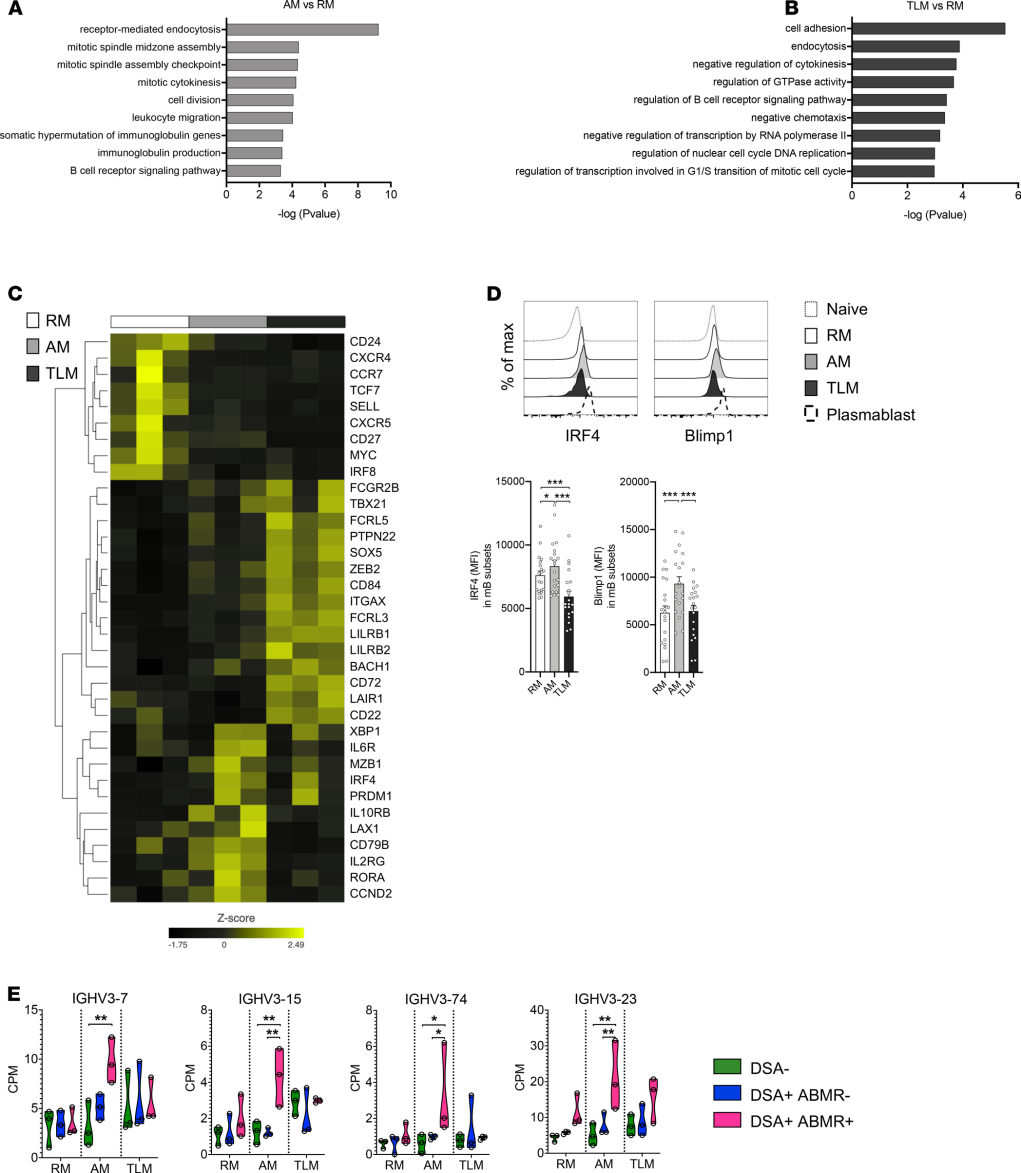
Transcriptional profiling of MBC subsets. RNA-Seq analysis of the 3 sorted MBC subsets was performed in 3 patients per group; DSA^–^ (*n* = 3), DSA^+^ABMR^–^ (*n* = 3), and DSA^+^ABMR^+^ (*n* = 3). GO analysis of differentially expressed genes (DEGs) in AM versus RM (**A**), or TLM versus RM subsets (**B**), from DSA^+^ABMR^+^ group (*n* = 3). (**C**) Heatmap generated by hierarchical clustering of selected genes in AM, RM, and TLM subsets from DSA^+^ABMR^+^ group (*n* = 3). (**D**) Representative examples of flow cytometry histograms and bar plots of MFI values of IRF4 and Blimp1 expression in naive (CD27^–^CD21^+^IgD^+^), RM, AM, TLM, and plasmablast (CD24^–^CD38^hi^) subsets from DSA^+^ABMR^+^ group (*n* = 20). Repeated measures 1-way ANOVA with Tukey’s correction. *P < 0.05; ***P < 0.001. Each dot represents 1 subject and horizontal lines of bars are mean values ± SEM. (**E**) Violin plots showing the expression levels of selected V_H_ germ line genes in AM, RM, and TLM subsets from indicated patient groups; DSA^–^ (*n* = 3), DSA^+^ABMR^–^ (*n* = 3), and DSA^+^ABMR^+^ (*n* = 3). Kruskal-Wallis with Dunn’s posttest. *P < 0.05; **P < 0.01. Each dot represents 1 subject and horizontal lines are median values ± SEM. CPM, counts per million; GO, Gene Ontology.

We next undertook RNA-Seq analyses of sorted RM, AM, and TLM subsets (Supplemental Figure 6) to analyze the transcriptional programs underlying the phenotypic states of each MBC subset in DSA^+^ABMR^+^ patients at the time of rejection. When compared with RM cells, the AM gene expression programs were significantly enriched in molecular pathways associated with B cell effector functions, including cell activation, cell division, somatic hypermutation, and Ig production (Figure 3A and Supplemental Table 4). In striking contrast, TLM cells were enriched in molecular pathways reflecting negative regulation of B cell activation and cell division (Figure 3B and Supplemental Table 5). *TBX21*, which encodes T-bet, was expressed in both AM and TLM cells, but at higher levels in the latter, consistent with the flow cytometry data (Figure 3C). However, compared with TLMs, AM cells uniquely upregulated *IL2RG*, reflecting their increased potential to respond to IL-21, and displayed elevated expression of the plasma cell genes *IL6R*, *MZB1*, and *XBP1* (Figure 3C). Importantly, interferon regulatory factor 4 (IRF4) and Blimp1, which are key transcription factors required for plasma cell differentiation in response to IL-21 (32), were expressed at higher levels at both transcript and protein levels in AM cells (Figure 3, C and D). Analysis of putative promoter/enhancer regions of DEGs that were delineated by a comparison of AM with RM cells from DSA^+^ABMR^+^ patients, using HOMER for matching T-bet binding motifs (–1000, +150 bps flanking transcription start site, TSS), revealed that 308 DEGs contained *TBX21* motifs around their TSS, with some genes containing multiple sites. Of genes containing *TBX21* binding motifs, 176/308 (57.1%) genes were upregulated in AM cells, while 132/308 (42.9%) were downregulated. Of note, *TBX21* motif–containing DEGs included *FCRL3*, *FCRL5*, *JCHAIN*, *MCL1*, *TCF7*, and *IL7R*, all of which were upregulated in AM cells (data not shown). TLM cells upregulated numerous genes associated with B cell exhaustion (*LILRB1*, *LILRB2*) and inhibitory function (*FCRL3*, *FCRL5*, *CD72*, *FCG2RB*, *PTPN22*; ref. 23), while RM cells expressed genes related to quiescence and central memory state (*SELL*, *CCR7*, *TCF7*; Figure 3C).

As organ rejection is known to involve the expansion of dominant B cell clones with characteristic *IGHV* usage (33, 34), we examined the differential expression of V_H_ germ line (*IGHV*) genes in RM, AM, and TLM subsets in DSA^+^ABMR^+^ patients. Strikingly, AM and TLM subsets from DSA^+^ABMR^+^ patients significantly differed from the DSA^+^ABMR^–^ and DSA^–^ groups (Supplemental Figure 7, A and B) by the selective expression of specific *IGHV* genes, reflecting a distinct *IGHV* gene usage (Table 1 and Supplemental Table 6). Notably, the elevated expression of the *IGHV3-7*, *IGHV3-15*, and *IGHV3-74* genes was selective to the AM subset in DSA^+^ABMR^+^ patients, while *IGHV3-23* was also elevated in RM and TLM subsets, albeit to a lesser extent (Figure 3E). In addition to differential *IGHV* usage, TLM cells from DSA^+^ABMR^+^ patients were enriched for *ZBTB32*, *DOK3*, and *FCRL4* (Supplemental Figure 7B), reflecting a more pronounced inhibitory state as compared with TLM cells from DSA^+^ABMR^–^ and DSA^–^ groups. Thus, the transcriptional state of AM cells in ABMR patients is readily distinguishable from that of their TLM and RM counterparts and suggests that many of these cells are poised to undergo differentiation into plasma cells in response to IL-21 signaling. Furthermore, the increased expression of selective *IGHV* genes within the AM compartment suggests expansion of dominant clones associated with ABMR.

### AM cells can be induced by CD40L and IL-21 stimulation in vitro.

Given that AM cells in DSA^+^ABMR^+^ patients displayed upregulated CD40 and IL-21R, we tested if CD40L and IL-21, which are T_FH_-derived signals, could promote their generation in vitro. Naive B cells from HCs were isolated (Supplemental Figure 6) and stimulated with a combination of signals including anti-IgM for 5 days (Supplemental Figure 8A). While anti-IgM and soluble CD40L (sCD40L) resulted in increased levels of T-bet in naive B cells, IL-21 was required for its maximum expression (Supplemental Figure 8B). The combination of IL-21 with anti-IgM and sCD40L resulted in higher induction of CD27^+^ among T-bet^+^ cells (Supplemental Figure 8C). IL-21 was also necessary for optimal activation of B cells (CD71^+^) and their maximal expression of CD11c and IL-21R. These T-bet^+^CD27^+^CD11c^+^IL-21R^+^ cells closely resembled AM cells present in blood of DSA^+^ABMR^+^ patients (Supplemental Figure 8D). The ability to induce T-bet expression was not unique to IL-21, as IFN-γ was also able to do so (Supplemental Figure 9, A and B). Notably, however, when combined with anti-IgM and sCD40L, IFN-γ preferentially induced T-bet^+^ cells that were CD27^–^, thus resembling TLM cells (Supplemental Figure 9C). Unlike IL-4 and IL-17, IFN-γ could also induce expression of CD11c and IL-21R, albeit at a lesser extent than IL-21 (Supplemental Figure 9D). Thus, compared with IFN-γ, IL-21 is a stronger inducer of AM-like cells in the presence of CD40-mediated stimulation. These data, along with the aforementioned analyses and our earlier study (6), suggest that the AM cells detected in DSA^+^ABMR^+^ patients likely arise from coordinated interactions with cognate T_FH_ cells in the context of allograft responses.

### AM cells are responsive to circulating T_FH_ cells and differentiate into DSA-producing plasma cells in an IL-21–dependent manner.

**Figure 4:**
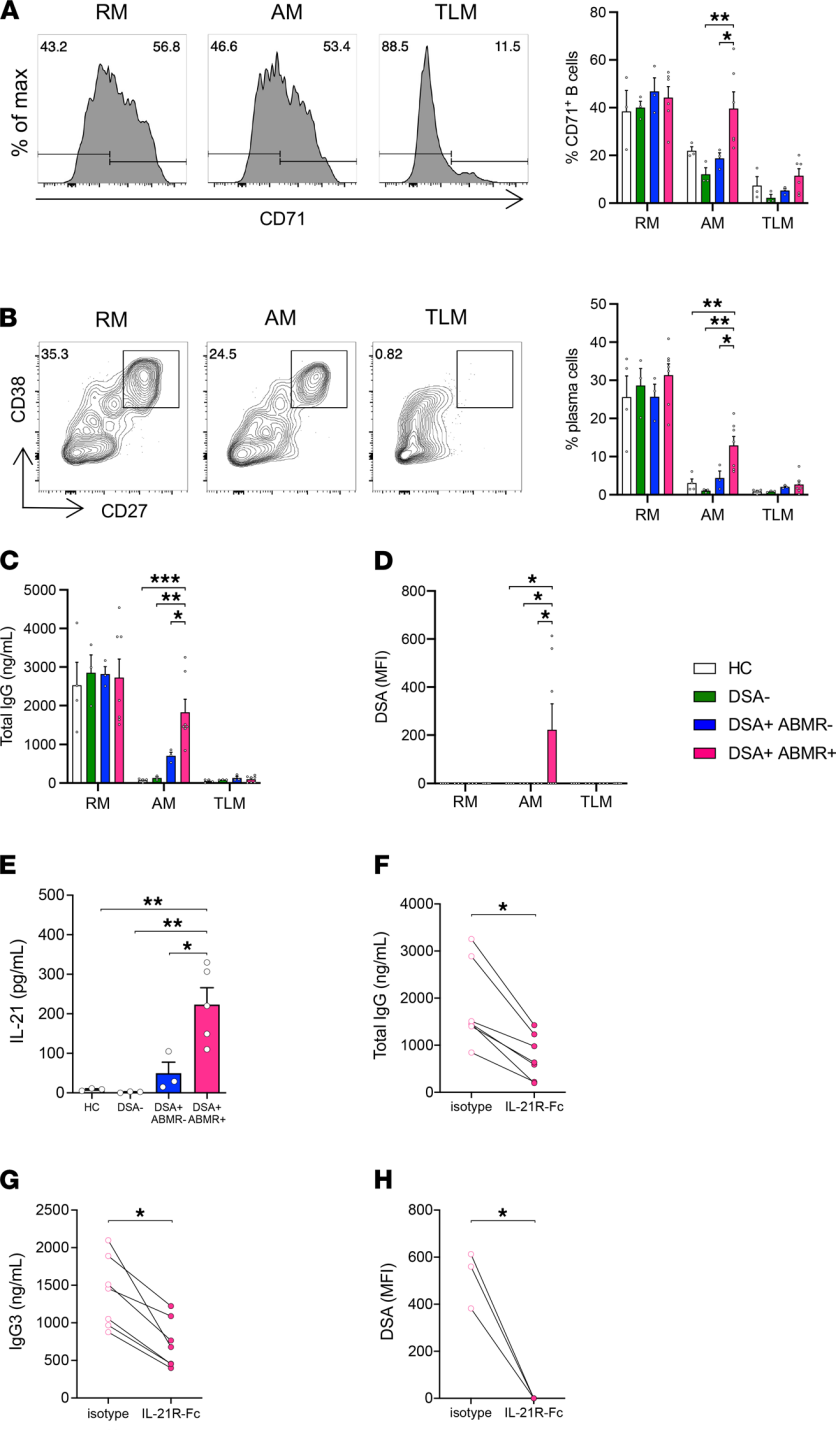
Analysis of cocultures of MBC subsets with autologous cT_FH_ cells. Coculture of the sorted MBC subsets with autologous cT_FH_ (CD3^+^CD4^+^CD45RO^+^CXCR5^+^) cells in the presence of SEB (6 days). (**A**) Representative examples of flow cytometry histograms and bar plots of percentages of CD71^+^ cells in RM, AM, and TLM cocultures after 6 days are displayed; HC (*n* = 3), DSA^–^ (*n* = 3), DSA^+^ABMR^–^(*n* = 3), and DSA^+^ABMR^+^ (*n* = 6) patients. (**B**) Representative examples of flow cytometry histograms and bar plots of percentages of CD27^+^CD38^+^ plasma cells in RM, AM, and TLM cocultures after 6 days are displayed; HC (*n* = 4), DSA^–^ (*n* = 3), DSA^+^ABMR^–^ (*n* = 3), and DSA^+^ABMR^+^ (*n* = 7) patients. (**C**) ELISA analysis of total IgG in supernatants of RM, AM, and TLM cocultures after 6 days is displayed; HC (*n* = 4), DSA^–^ (*n* = 3), DSA^+^ABMR^–^ (*n* = 3), and DSA^+^ABMR^+^ (*n* = 7) patients. (**D**) Luminex analysis of DSAs in supernatants of RM, AM, and TLM cocultures after 6 days is displayed; HC (*n* = 4), DSA^–^ (*n* = 3), DSA^+^ABMR^–^ (*n* = 3), and DSA^+^ABMR^+^ (*n* = 7) patients. (**E**) Cytometric bead array analysis of IL-21 in supernatants of AM cocultures after 6 days is displayed; HC (*n* = 3), DSA^–^ (*n* = 3), DSA^+^ABMR^–^ (*n* = 3), and DSA^+^ABMR^+^ (*n* = 5) patients. IL-21R–Fc or isotype-matched control were added to AM cocultures of DSA^+^ABMR^+^ (*n* = 7) patients. Total IgG (**F**), IgG3 (**G**), and DSA (**H**) in supernatants of AM cocultures were measured after 6 days. Kruskal-Wallis with Dunn’s posttest for panels **A**–**E**. Wilcoxon’s matched pairs signed rank test for panels **F**–**H**. *P < 0.05; **P < 0.01; ***P < 0.001. Each dot represents 1 subject and horizontal lines of bars are mean values ± SEM. SEB, staphylococcal enterotoxin B.

To directly test the above hypothesis, we analyzed the effect of autologous T_FH_ cells in promoting the differentiation of AM cells into plasma cells that secrete DSAs. This involved use of a 6-day coculture system with sorted circulating T_FH_ (cT_FH_) and MBC subsets from individual patients and stimulating the former with SEB. We note that AM cells did not differentiate into plasma cells in the absence of cT_FH_ cells or SEB in cocultures (data not shown). AM cells from DSA^+^ABMR^+^ patients manifested enhanced activation (CD71^+^) and more pronounced differentiation into plasma cells as compared with those from DSA^–^ or DSA^+^ABMR^–^ patients and HCs (Figure 4, A and B). Accordingly, the amounts of total IgG produced by AM cells were substantially higher in DSA^+^ABMR^+^ patients (Figure 4C). Importantly, antigen-specific IgGs (DSAs) were specifically detected only in cocultures of AMs with cT_FH_ cells in these patients but not in the DSA^+^ABMR^–^ group (Figure 4D). This indicates an enrichment for donor antigen-specific cells in AM cells from DSA^+^ABMR^+^ patients as compared with those of the DSA^+^ABMR^–^ group. The production of IgGs and DSAs by AM cells was regulated by IL-21 signaling, as the levels of T_FH_-derived IL-21 in the cocultures were increased in DSA^+^ABMR^+^ patients, and conversely the inhibition of IL-21 signaling resulted in significant reduction of secreted IgGs, including IgG3 subclass, and DSAs (Figure 4, E–H). We note that TLM cells failed to differentiate into plasma cells and generate DSAs. Thus, AM cells from DSA^+^ABMR^+^ patients manifested increased capacities to undergo activation and differentiation into plasma cells through their interactions with T_FH_ cells in an IL-21–dependent manner. Furthermore, they contained alloreactive clones, which when activated differentiated into plasma cells that secreted DSAs.

### Magnitude and dynamics of AM cell expansion correlate with ABMR manifestations and timing.

**Figure 5:**
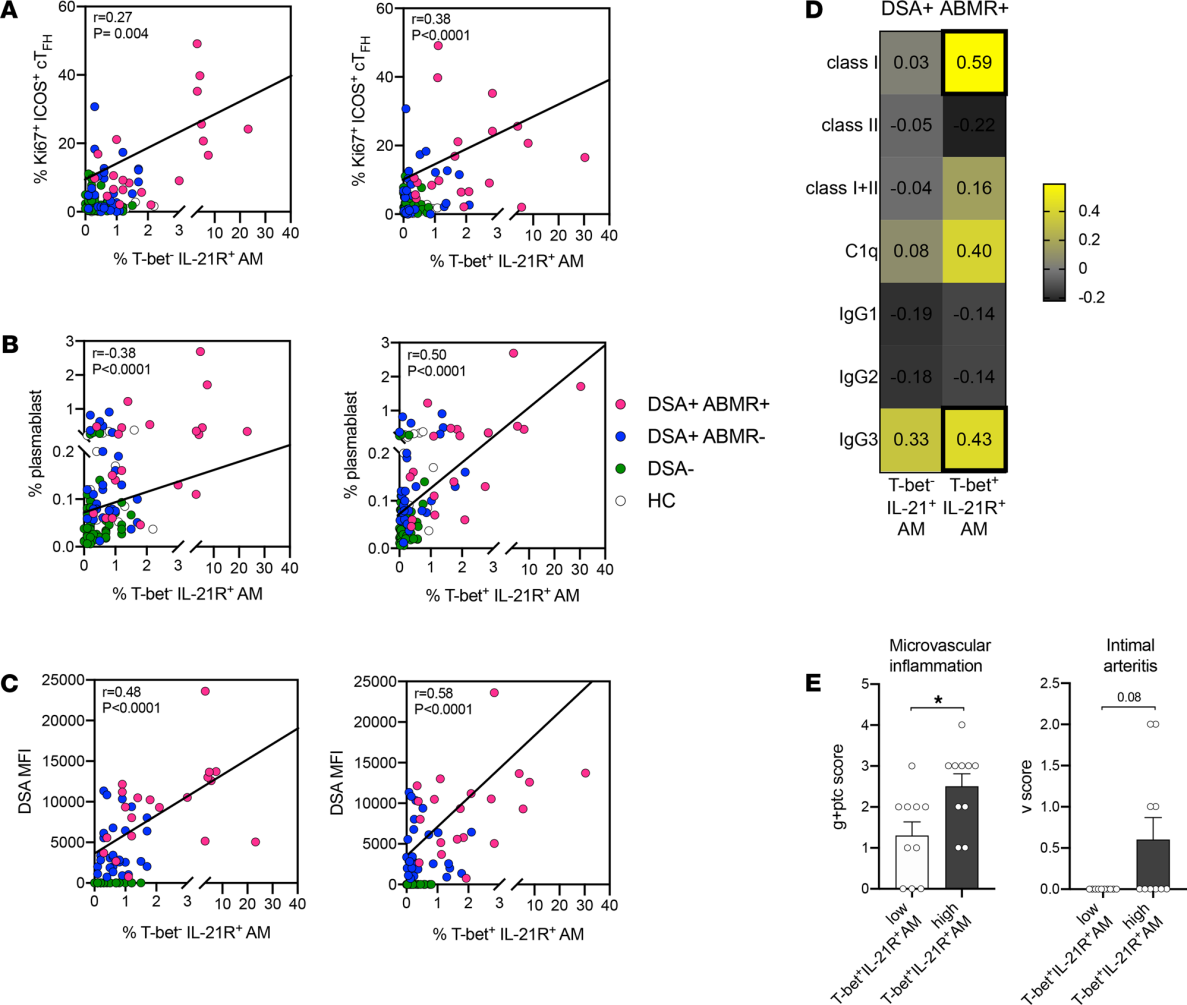
Correlation of frequencies of AM subsets with disease manifestations of ABMR. Spearman’s correlation analysis of percentages of T-bet^–^IL-21^+^ AM and T-bet^+^IL-21^+^ AM subsets with percentages of blood (**A**) Ki67^+^ICOS^+^ cT_FH_ (CD3^+^CD4^+^CD45RO^+^CXCR5^+^) cells, (**B**) plasmablasts (CD19^+^CD24^–^CD38^hi^), and (**C**) DSA MFI levels measured in serum by Luminex; HC (*n* = 17), DSA^–^ (*n* = 48), DSA^+^ABMR^–^ (*n* = 28), and DSA^+^ABMR^+^ (*n* = 20). (**D**) Heatmap showing Spearman’s correlation coefficients of percentages of T-bet^–^IL-21^+^ AM and T-bet^+^IL-21^+^ AM subsets with MFI levels of class I, class II, sum of class I plus II, C1q-binding, and IgG subclasses of DSAs measured in serum from DSA^+^ABMR^+^ patients, by Luminex. Bold squares indicate correlations with *P* < 0.05. DSA class I and II analyses were performed for *n* = 20, DSA IgG subclass analysis was performed for *n* = 18, and DSA C1q-binding analysis was performed for *n* = 19 patients. (**E**) DSA^+^ABMR^+^ patients were stratified into 2 subgroups based on the median percentage of T-bet^+^IL-21^+^AM cells less than 1.79% (low) and more than 1.79% (high) in the DSA^+^ABMR^+^ group. Histological Banff scores of kidney allograft lesions were evaluated at the time of ABMR; microvascular inflammation = g + ptc Banff score and intimal arteritis = v Banff score. Mann-Whitney *U* test. *P < 0.05. Each dot represents 1 subject and horizontal lines of bars are mean values ± SEM.

We next evaluated whether the frequencies of AM cells correlated with the amplitude of blood cT_FH_, plasmablast, and DSA responses. AM frequencies significantly correlated with those of activated Ki67^+^ICOS^+^ cT_FH_ and plasmablasts (Supplemental Figure 10, A and B). Also, higher DSA levels in serum samples paralleled increased AM frequencies, further suggesting that expansion of these cells reflects the strength of the donor-specific response in vivo (Supplemental Figure 10C). Furthermore, AM frequencies significantly correlated with IgG3 DSA levels in DSA^+^ABMR^+^ patients (Supplemental Figure 10D). These correlations between AM cells with cT_FH_, plasmablasts, and DSAs were stronger for T-bet^+^IL-21R^+^ cells as compared with the T-bet^–^IL-21R^+^ AM subset (Figure 5, A–D). Importantly, DSA^+^ABMR^+^ patients with high T-bet^+^IL-21R^+^ AM frequencies (>1.79%) also manifested more microvascular inflammation and intimal arteritis lesions (Figure 5E).

We next evaluated the relationship between the dynamics of the AM cell response and the timing to ABMR onset posttransplant. We found that patients with late (after 3 months posttransplant) DSA^+^ABMR^+^ displayed significantly more T-bet^+^IL-21R^+^ AM cells than those with early (before 3 months) DSA^+^ABMR^+^ (Supplemental Figure 11A). Notably, T-bet^+^IL-21R^+^ AM cells were predominantly IgD^+^ in the late DSA^+^ABMR^+^ group, while being mostly IgD^–^ in the early forms (Supplemental Figure 11B). We next performed longitudinal analyses of samples collected pretransplant and at 1, 3, 6, and 12 months posttransplant. Both IgD^+^ and IgD^–^ T-bet^+^IL-21R^+^ AM cells were present at low frequencies pretransplant and were induced posttransplant (Supplemental Figure 11C). Importantly, the dynamics of IgD^+^ cell expansion paralleled with late onset of DSA^+^ABMR^+^, while that of IgD^–^T-bet^+^IL-21R^+^ AM cells coincided with early DSA^+^ABMR^+^ occurrence. Consistent with this timing, IgD^–^ cells contained more proliferating (Ki67^+^) cells than IgD^+^T-bet^+^IL-21R^+^ AM cells (Supplemental Figure 11D). Clustering analyses confirmed the higher frequencies of the IgD^+^T-bet^+^IL-21R^+^ cells (cluster 11) in late DSA^+^ABMR^+^, compared with predominance of the IgD^–^T-bet^+/–^IL-21R^+/–^ cells (clusters 4 and 7) in early forms (Supplemental Figure 12, A and B). We note that the 3 DSA^+^ABMR^+^ patients with pure ABMR displayed similar distribution of clusters 4, 7, and 11 to the other 17 patients with mixed ABMR (Supplemental Figure 12A). Thus, the temporal dynamics of expansion of the AM subset, enriched in T-bet^+^IL-21R^+^ cells, coincided with the timing of ABMR onset posttransplant, and their increased frequencies were associated with the severity of ABMR manifestations.

### AM cells and their restricted IGHV sequences are detected within kidney allografts of patients with ABMR.

**Figure 6:**
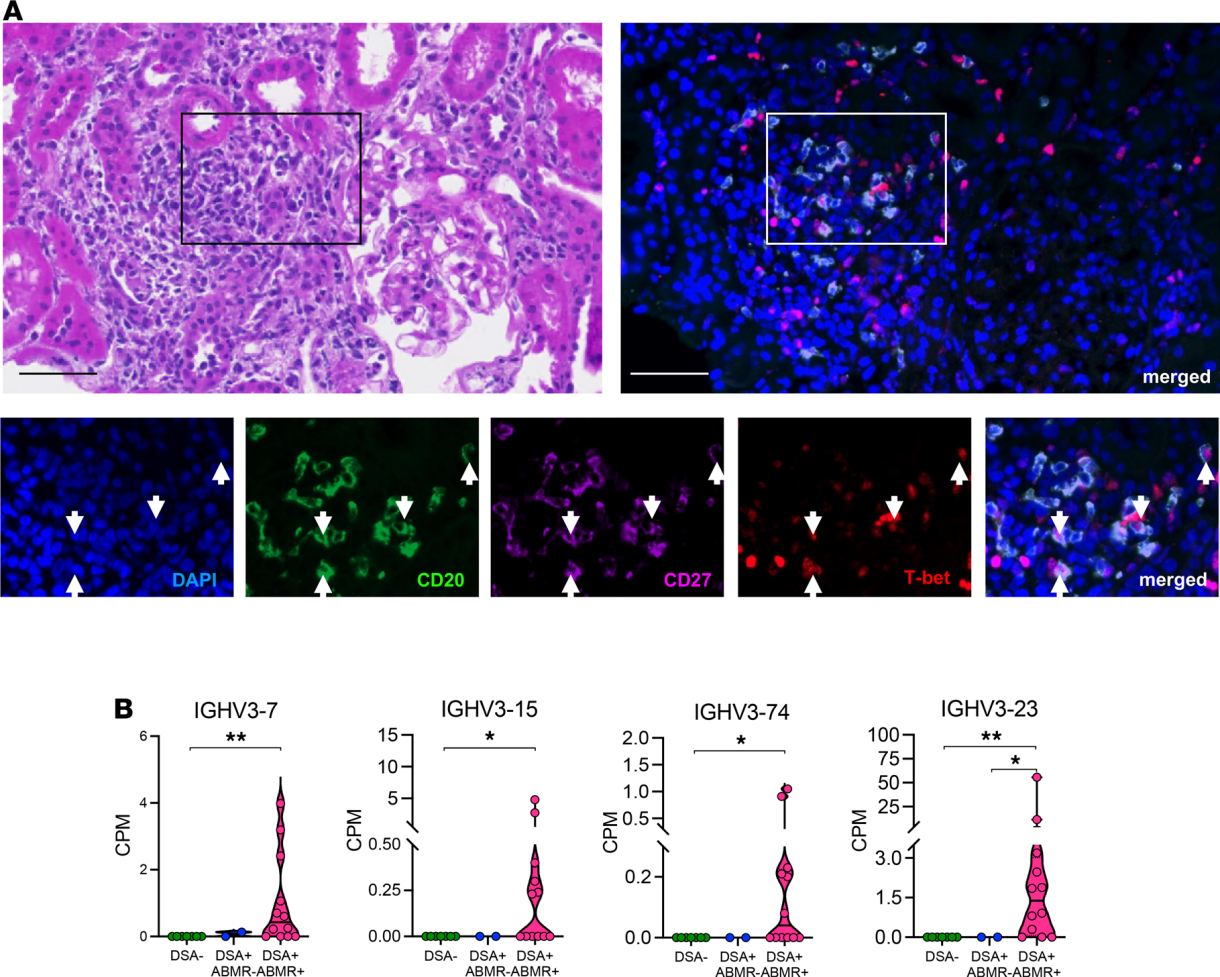
AM cells and their restricted *IGHV* sequences within kidney allografts of patients with ABMR. (**A**) Representative multiplex immunofluorescence staining performed on a kidney allograft biopsy from a patient with acute DSA^+^ABMR^+^, at the time of ABMR episode. Arrows indicate CD20^+^CD27^+^T-bet^+^ (triple-positive) cells. Scale bars indicate 50 μm. (**B**) RNA-Seq analysis of kidney allograft biopsies was performed; DSA^–^ (*n* = 7), DSA^+^ABMR^–^ (*n* = 2), and DSA^+^ABMR^+^ (*n* = 12). Violin plots showing the expression levels of selected *IGHV* genes in kidney allograft biopsies. Kruskal-Wallis with Dunn’s posttest. *P < 0.05; **P < 0.01. Each dot represents 1 subject and horizontal lines are median values ± SEM.

Using multiplex immunofluorescence staining, we investigated whether AM cells could be detected within kidney allografts of patients. We detected the presence of CD20^+^CD27^+^T-bet^+^ cells, consistent with an AM phenotype, within the interstitial inflammatory infiltrate of allografts from patients with acute (Figure 6A) or chronic (Supplemental Figure 13) forms of DSA^+^ABMR^+^, and these cells represented 1 ± 0.8 cells/mm^2^ of tissue. We did not detect any of these cells in allografts from DSA^+^ABMR^–^ or DSA^–^ patients (Supplemental Figure 13). We next determined whether the AM-specific molecular signatures, including select *IGHV* genes, previously defined in circulating AM cells were also found within kidney allografts. We therefore recruited 21 additional kidney transplant patients from our Transplant Institute with available allograft biopsy samples (Supplemental Table 7) and performed RNA-Seq analysis on these samples (DSA^–^ *n* = 7, DSA^+^ABMR^–^ *n* = 2, DSA^+^ABMR^+^ *n* = 12). The RNA-Seq profile of allografts from DSA^+^ABMR^+^ patients markedly differed from that of DSA^+^ABMR^–^ and DSA^–^ groups (Supplemental Figure 14A). This distinct transcriptional profile was due to the increased expression of the hallmark molecular features of AM cells *TBX21* (T-bet), *MS4A1* (CD20), *CD27*, and *IL21R* in DSA^+^ABMR^+^ patients compared with the other patient groups (Supplemental Figure 14B). Additionally, 6 of the 9 AM-specific *IGHV* genes, including *IGHV3-7*, *IGHV3-15*, and *IGHV3-74*, previously found to be increased in circulating AM cells (Figure 3E), were also significantly upregulated in allografts from DSA^+^ABMR^+^ patients (Figure 6B). We note that *IGHV3-23* and 2 of the 3 TLM-specific *IGHV* (*IGHV1-69*, *IGHV3-11*) genes were also upregulated in allografts of these patients as compared with the DSA^–^ group (Table 1). Thus, circulating AM cells and their molecular signatures could be detected within allografts of patients undergoing ABMR.

## Discussion

The phenotypic states and functional roles of T-bet–expressing, antigen-experienced B cells have previously been analyzed in multiple clinical settings with a focus on their protective responses in viral and bacterial infections and their pathogenic potential in autoimmune diseases but not in organ transplantation (35, 36). This study uncovers the emergence of these cells in the context of pathogenic alloimmune responses directed against organ transplants. As noted in other disease settings in which T-bet–expressing B cell responses have been analyzed, alloimmunity in organ transplantation is dominated by a sustained type 1 (IFN-γ) and T_FH_ (IL-21) cell-driven inflammatory environment (27, 37). Consistent with studies in other disease contexts, the T-bet–expressing B cells in ABMR patients were heterogeneous and comprised expanded AM as well as TLM cells. However, our extensive phenotypic, molecular, and functional analyses strongly suggest that the T-bet–expressing AM cells, unlike TLM cells, are the source of pathogenic alloreactive humoral responses in ABMR.

We provide the following lines of evidence that T-bet–expressing AM B cells are pathogenic drivers in patients undergoing ABMR and can be functionally distinguished from their TLM cell counterparts: (a) AM cells express higher levels of the B cell costimulatory receptors IL-21R and CD40, (b) manifest increased expression of IRF4 and Blimp1 that are required for plasma cell differentiation, and (c) proliferate and display amplification of restricted *IGHV* sequences consistent with their clonal expansion in vivo during ABMR; (d) AM cells from ABMR patients preferentially differentiate into plasma cells when cocultured with autologous T_FH_ cells in an IL-21–dependent manner and (e) uniquely generate DSAs; (f) temporal dynamics of expansion of AM cells coincide with early ABMR involving preformed DSAs and with late ABMR associated with de novo–generated DSAs; (g) AM cell frequency is correlated with the pathogenic IgG3 DSA isotype involved in ABMR and is predictive of the severity of histological lesions; and (h) AM cells were detected within kidney allografts of ABMR patients, along with their characteristic amplified *IGHV* sequences.

While AM and TLM cells share the expression of T-bet and IL-21R, fundamental differences exist in their differentiation states and effector functions. AM cells are heterogeneous, preferentially expressing the key costimulatory receptor CD27, which is acquired through germinal center (GC) responses (38, 39). It is therefore likely that the majority of AM cells represent recent GC emigrants. Moreover, most T-bet^+^ AM cells from early ABMR (sensitized patients) lacked IgD, suggestive of switched memory B cells, while T-bet^+^ AM cells from late ABMR (de novo DSA patients) displayed elevated frequencies of unswitched IgD^+^ memory B cells that likely reflect an earlier stage in the alloreactive humoral response. Notably, AM cells appear poised for plasma cell differentiation (14). Our RNA-Seq and flow cytometry analyses suggest a molecular basis for the poised effector state of AM cells because they expressed higher levels of the transcription factors IRF4 and Blimp1, which are required for plasma differentiation (32, 40, 41), as well as increased *IL6R*, *MZB1*, and *XBP1*, 3 key genes modulating plasma cell differentiation (14, 42). Analysis of putative promoter/enhancer regions from AM cells of DSA^+^ABMR^+^ patients suggests that T-bet (*TBX21*) directly binds to promoter regions of a large set of genes in AM cells and either activates or represses their transcription. We note that AM cells expressed low levels of CD38 and high levels of CD20, thereby supporting our overall conclusion that these cells represent plasma cell precursors in the context of pathogenic alloimmune responses. Therefore, concomitant targeting of the AM cell compartment and their plasma cell progeny could provide an optimal means of dampening the humoral response during ABMR.

Strikingly, AM cells were selectively enriched for IgH transcripts containing V_H_ germ line sequences that have been previously documented to predominate during organ rejection, including *IGHV3-7*, *IGHV3-15*, *IGHV3-74*, and *IGHV3-23* (33, 43, 44). This specific *IGHV* gene usage likely reflects responses to a common pool of highly immunogenic alloantigens and potentially auto- and recall-antigens. These could induce the emergence of dominant B cell clones of varying specificities that may drive organ rejection. While B cell clones with dominant *IGHV3-7*, *IGHV3-15*, *IGHV3-74*, and *IGHV3-23* germ line genes were previously detected in blood of patients with organ rejection, our study reveals that these clones appear differentially distributed among B cell subsets. The *IGHV3-23* gene, which is documented across several studies (33, 43–45), involved in superantigen recognition, was found to be expressed by the 3 MBC subsets (RM, AM, and TLM cells). In contrast the *IGHV3-7*, *IGHV3-15*, and *IGHV3-74* genes were unique to AM cells. Importantly, our study is the first to our knowledge to document a memory subset–specific (AM-specific) V_H_ gene usage, in circulating and allograft-infiltrating B cells in patients undergoing ABMR. It is likely that these infiltrating AM cells bearing pathogenic *IGHV* genes may participate in the DSA production in situ in kidney allografts. In contrast, AM cells from DSA^+^ABMR^–^ patients, which lacked these pathogenic *IGHV* genes, were less likely to produce DSAs in vitro when stimulated with cT_FH_ cells. Thus, this strongly suggests the deleterious role of specific B cell clones within the AM cell compartment during ABMR and suggests their value for immune monitoring as predictive biomarkers for ABMR. However, it remains to be resolved whether AM cell function and pathogenic potential are similar according to the timing (early versus late) of ABMR occurrence and its clinical presentation (pure versus mixed ABMR).

TLM cells in ABMR patients were substantially different from their AM cell counterparts, as they displayed lower levels of the costimulatory molecules CD27 and CD40 and were hyporesponsive to T_FH_ cell help. TLM cells have been recently shown to be localized in the extrafollicular zone of lymph nodes and virtually absent from the lymphatic circulation (26). It is tempting to speculate that TLM cells are mainly generated through the extrafollicular pathway, as they have been shown to display poor affinity maturation, to generate short-lived plasma cells, and to strongly respond to TLR stimulation and IFN-γ, a cytokine mainly found outside GCs (46). On the other hand, the common *IGHV3-23* gene usage between AM and TLM cells and the T-bet^hi^ expression of TLM cells compared with the T-bet^intermediate^ status of AM cells suggest that these 2 subsets may be partly clonally related and that TLM cells may arise from further differentiation of AM cells under persistent antigenic stimulation. As with exhausted CD8^+^ T cells, these exhausted like B cells lose classical markers of effector cells and acquire an inhibitory expression profile, including CD72 and the inhibitory IgG receptors CD32b and FcRL5 that are associated with impaired effector function (47).

One of the unexpected findings of this study was the expansion of TLM cells during ABMR, coincident with AM cells. Given their functional properties, TLM cells would be expected to be generated at low levels during a highly efficient antibody response such as in ABMR. A likely explanation could be that these cells arise concomitantly to counterbalance the hyperactivation state of their AM counterparts in the context of chronic activation. Indeed, TLM cells during HIV infection were reported to be involved in a regulatory loop triggered by the binding of circulating IgG3 and C1q (also present in excess during ABMR) onto their surface IgM, which conveyed a strong B cell receptor–mediated inhibitory signal to these cells (48).

IL-21 is the principal cytokine of T_FH_ cells and is a major regulator of B cell–mediated immunity. It induces potent B cell activation, drives their differentiation into plasma cells, and favors the generation of class-switched antibodies of IgG3 isotype (49, 50). We and others have shown that IL-21 is a major cytokine produced by donor-specific cT_FH_ cells and that increased IL-21 production is predictive of ABMR (6, 27, 28, 51). Additionally, IL-21R blockade results in reduced plasma cell differentiation in vitro in a coculture model of T_FH_-B cells stimulated with donor antigen, and it could delay skin allograft rejection in vivo in mice (52, 53). Here, we show that AM cells specifically upregulated IL-21R in ABMR and that IL-21 was required for the differentiation of these cells into plasma cells that secrete DSAs. Importantly, IL-21R, along with T-bet, were induced on naive B cells upon their activation by IL-21 and CD40 stimulation. Thus, in concert with our earlier study demonstrating the emergence of cT_FH_ cells and increased IL-21 production in patients undergoing ABMR (6), we now delineate a pivotal role for AM B cells in promoting the pathogenic humoral responses. In so doing, we propose alloreactive AM B cells and the IL-21 pathway as novel therapeutic targets to promote the durability of allografts in organ transplantation.

## Methods

### Study design.

This study was performed on samples from patients who underwent kidney transplantation between January 2013 and December 2017 at University of Pittsburgh Medical Center and who were recruited to participate in a biorepository initiative at Thomas E. Starzl Transplantation Institute (STI). All patients signed a written informed consent document (IRB PRO12030552; PRO17020318).

A total of 530 patients were screened for the following immunological events: presence of posttransplant DSA and biopsy-proven ABMR. We identified 48 patients who developed DSA in the first 24 months posttransplant and had available PBMCs, defining 2 study groups: patients with ABMR (DSA^+^ABMR^+^, *n* = 20) and patients without ABMR (DSA^+^ABMR^–^, *n* = 28). Forty-eight age- and sex-matched patients with no DSA or ABMR (DSA^–^) in the first 24 months posttransplant were selected to form the third study group. In addition, 17 age- and sex-matched HCs from the STI Human Immunology Program were also enrolled. The flow chart of the study design is depicted in Supplemental Figure 1A. Clinical data of the study patients were extracted from the prospective database of the STI biorepository.

### Blood samples.

PBMCs and sera were prospectively collected and banked at pretransplant; at 1, 3, 6, 12, and 24 months posttransplant; and at the time of clinically indicated kidney allograft biopsies. The presence of DSAs in sera was systematically assessed at these time points. Surveillance protocol allograft biopsies were performed at 3 and 12 months posttransplant (Supplemental Figure 1B). We analyzed cross-sectional PBMCs and serum samples banked from the blood collected at the time of the immunological events of interest: (a) detection of posttransplant DSA for DSA^+^ABMR^–^ patients and (b) detection of ABMR in the presence of DSA for DSA^+^ABMR^+^ patients. In DSA^–^ patients, the blood samples were analyzed at the time of a protocol biopsy, and their time points were matched with those from DSA^+^ABMR^–^ and DSA^+^ABMR^+^ patients (Supplemental Figure 1B and Supplemental Table 2). Patients for whom cryopreserved PBMC samples were available at time points considered (pretransplant and 1, 3, 6, and 12 months posttransplant) were included in the longitudinal flow cytometry analysis in Supplemental Figure 11C.

### Detection and characterization of DSAs.

The presence of anti-HLA antibodies with known reactivity against the donor HLA molecules (DSAs) in sera was systematically assessed at indicated time points described above. Pan-IgG anti-HLA -A, -B, -C, -DRB1/3/4/5, -DQB1, -DQA1, and -DPB1 DSAs were assessed in sera using single-antigen flow bead (SAB) assays (One Lambda, Thermo Fisher Scientific) on a Luminex platform (Luminex Corp.) according to manufacturer’s protocol. DSA^+^ sera were tested for the presence of C1q binding using the C1q-modified SAB assay (One Lambda, Thermo Fisher Scientific), and IgG subclasses were tested using the SAB assay, substituting PE-conjugated anti-human IgG1 (clone HP6001), IgG2 (clone 31-7-4), IgG3 (clone HP6050), and IgG4 (clone HP6025) secondary antibodies for anti-human IgG from SouthernBiotech. Normalized MFI cutoffs for positive results were MFI > 1000 for the pan-IgG and IgG subclass assays and MFI > 500 for the C1q assays (5).

### Kidney allograft histology.

Kidney allograft tissues were fixed in formalin and stained with Masson’s trichrome and periodic acid–Schiff. Allograft biopsies were scored and graded from 0 to 3, and diagnosis of ABMR was histologically defined using the international Banff 2017 criteria and was reviewed by an expert clinical transplant pathologist. Lesions of T cell–mediated rejection (TCMR) were also defined according to Banff criteria (54). DSA^–^ and DSA^+^ABMR^–^ patients did not show signs of ABMR or TCMR at the time of sampling. All 20 ABMR cases were acute and C4d-positive, with the exception of 1 case of chronic active ABMR (cg > 0) and 1 case of C4d-negative ABMR.

### Spectral flow cytometry.

Spectral flow cytometry is a fluorochrome-based system that allows analysis of protein expression of more than 20 parameters simultaneously with single-cell resolution and minimal signal overlap between channels. Full details of the antibodies used are given in Supplemental Table 8. Briefly, 1 to 2 million PBMCs were thawed and incubated with a mixture of antibodies diluted in 75% phosphate-buffered saline (PBS) and 25% Brilliant Violet Buffer (BD Biosciences) for 30 minutes at 4°C. Cells were surface-stained in Fc receptor blocking media (10% FCS PBS). Then PBMCs were washed, fixed, permeabilized with fixation/permeabilization buffer (eBioscience, Thermo Fisher Scientific) for 40 minutes at 4°C, washed with permeabilization buffer (eBioscience, Thermo Fisher Scientific), incubated in the dark for 30 minutes at 4°C with intracellular antibodies, and washed before acquisition on Aurora spectral flow cytometer (Cytek).

### High-dimensional flow cytometry data analysis.

The flow cytometry data were first curated with FlowJo software (Tree Star) to exclude debris, dead cells, and doublets, and MBCs were identified by gating for further downstream analyses. Single-cell data were normalized and analyzed simultaneously using Cytobank software (55). t-SNE analysis makes a pairwise comparison of cellular phenotypes to optimally plot similar cells close to each other and reduces multiple parameters into 2 dimensions (t-SNE X, t-SNE Y) (56). Data from Flow Cytometry Standard files were normalized, downsampled, and concatenated to create t-SNE maps. To run t-SNE algorithm, we applied the following settings: 3000 iterations, perplexity of 30, and θ of 0.5. Cell clusters were determined by the SPADE (57) algorithm using 12 as the target number of nodes without downsampling events. The cell clusters identified by SPADE were overlaid on the consensus t-SNE maps for visualization, and a heatmap was generated to delineate specific phenotypic patterns.

### Cell sorting.

PBMCs were thawed, stained, and sorted on a BD Biosciences FACSAria II cytometer. Naive B cells were sorted as CD3^–^CD19^+^CD38^lo^CD27^–^CD21^+^IgD^+^, RM cells as CD3^–^CD19^+^CD38^lo^CD27^+^CD21^+^, AM cells as CD3^–^CD19^+^CD38^lo^CD27^+^CD21^–^, TLM cells as CD3^–^CD19^+^CD38^lo^CD27^–^CD21^–^, and cT_FH_ as CD19^–^CD3^+^CD4^+^CD45RO^+^CXCR5^+^ cells.

### Cocultures.

Sorted cT_FH_ were cocultured with sorted autologous RM, AM, or TLM cells (2 × 10^4^) at 1:1 ratio with SEB (1 μg/mL, Toxin Technology) in RPMI (Gibco, Thermo Fisher Scientific) supplemented with 10% FCS, 100 IU/mL penicillin, 100 mg/mL streptomycin (Life Technologies, Thermo Fisher Scientific), 1 M HEPES buffer (Corning), and 2 mM l-glutamine. Some cocultures were supplemented with mouse IL-21R–Fc (R&D Systems, Bio-Techne) or isotype-matched control (R&D Systems, Bio-Techne). After 6 days of coculture, cells were stained with CD4, CD19, CD27, CD38, and CD71 antibodies before acquisition on the cytometer. DSAs were detected in supernatants after 6 days of coculture using Luminex SAB assay.

### IgG ELISA.

Total IgG production was measured in coculture supernatants using Human IgG total ELISA kit, and IgG3 production was measured with IgG Subclass Human ELISA kit (both eBioscience, Thermo Fisher Scientific).

### IL-21 assay.

Supernatants from cocultures were collected and analyzed for the presence of IL-21 by Cytometric Bead Array (BD Biosciences) according to the manufacturer’s protocol. All events were acquired using a Fortessa cytometer.

### B cell activation.

Sorted naive B cells (1 × 10^5^ in 200 μL per well) were plated and activated with goat anti-human IgM F(ab′)_2_ (10 μg/mL, Jackson ImmunoResearch), recombinant human CD40L (500 ng/mL, Enzo Life Sciences), IL-21 (100 ng/mL, Gibco, Thermo Fisher Scientific), IFN-γ (50 ng/mL, R&D Systems, Bio-Techne), IL-4 (25 ng/mL, R&D Systems, Bio-Techne), and IL-17 (50 ng/mL, R&D Systems, Bio-Techne). Cells were cultured in complete RPMI and incubated at 37°C for 5 days. Cells were harvested and stained with CD19, CD38, T-bet, CD27, CD11c, IL-21R, and CD71 antibodies before acquisition on a cytometer.

### RNA-Seq.

Total RNA was extracted from MBC subsets (RM, AM, and TLM cells) and FFPE sections of kidney allograft biopsies. RNA was isolated using miRNeasy Mini Kit (QIAGEN). cDNA synthesis and amplification were performed with SMARTer Stranded Total RNA-Seq Kit v2 — Pico Input Mammalian (Takara). Libraries were sequenced on an Illumina NextSeq 500 using 75 bp paired-end reads. The paired-end reads were checked for quality and adapters using FastQC (v0.11.7). These quality trimmed reads were later mapped against the Ensembl human reference genome (GRCh38 version 91) using HISAT2 mapper (v2.1.0). Counts for genes were generated using HT-Seq (v0.11.2) on the mapped files. Bioconductor R (v3.8) package EdgeR (v3.24.1) was used to analyze differential gene expression.

GO analyses were performed on DEGs between RM, AM, and TLM cells from DSA^+^ABMR^+^ patients (with *P* ≤ 0.054), using topGO (v2.34.0) in R using the human Ensembl database library EnsDb.Hsapiens.v86 (v2.99.0). The classic Fisher’s test with elimination algorithm using default *P* < 0.01 from topGO was applied to DEG sets to conditionally enrich for leaf/terminal nodes.

### Data sharing information.

Gene Expression Omnibus accession number for RNA-Seq: GSE155670.

### Multiplex immunofluorescence of kidney allografts.

FFPE blocks of kidney allograft biopsies from transplant patients were cut (4 μm), then underwent sequential rehydration and antigen retrieval in citrate pH 6 solution buffer. The sections were incubated overnight with anti-CD20 (mouse, M0755, Dako), anti-CD27 (armenian hamster, ab219779, Abcam), and anti–T-bet (rabbit, ab150440, Abcam) antibodies. After washing, the sections were incubated 30 minutes with secondary anti-mouse (AF488, Dako), anti–armenian hamster (AF568, Abcam), and anti-rabbit (Cy5, Abcam) antibodies. Nuclei were counterstained with DAPI, and slides were mounted using ProLong medium (Thermo Fisher Scientific). Sections were also stained with hematoxylin and eosin for histological evaluation. Each experiment was performed concomitantly with a positive control (section from kidney allograft removed due to incurable ABMR or human spleen) and a negative control (section from kidney allograft incubated with secondary antibody without primary antibody). Images were acquired on a Zeiss Axio Scan instrument. We used the number of CD20^+^CD27^+^T-bet^+^ (triple-positive) cells per 10 consecutive high-power fields for cell quantification.

### Statistics.

Mean ± SEM values and frequencies are provided for the description of the continuous and categorical variables, respectively. The means and proportions were compared using *t* test and χ^2^ test (or the Mann-Whitney *U* test and Fisher’s exact test if appropriate, respectively). Multiple groups were analyzed by Kruskal-Wallis test or 1-way ANOVA with Tukey’s post hoc test for adjustment for multiple comparisons. Values of *P* < 0.05 were considered statistically significant, and all tests were 2 sided. Analyses were performed using GraphPad Prism version 8, Cytobank (http://www.cytobank.org), Partek Flow (http://www.partek.com/partek-flow/), and R software (R Development Core Team).

### Study approval.

The study protocol was approved by the University of Pittsburgh IRB (IRB PRO12030552; PRO17020318). Subjects provided written informed consent prior to inclusion in the study.

## Author contributions

KL and DM designed the study; KL, EB, CM, and XG performed the experiments; KL, EB, LL, AC, BR, UC, GC, DL, and HS analyzed the data; CM and EB provided clinical data; PR provided the pathology data; AZ provided the tissue typing data and analyzed the DSA results; and KL, DM, HS, and CL drafted and revised the paper. All authors approved the final version of the manuscript.

## Supplementary Material

Supplemental data

## Figures and Tables

**Table 1:**
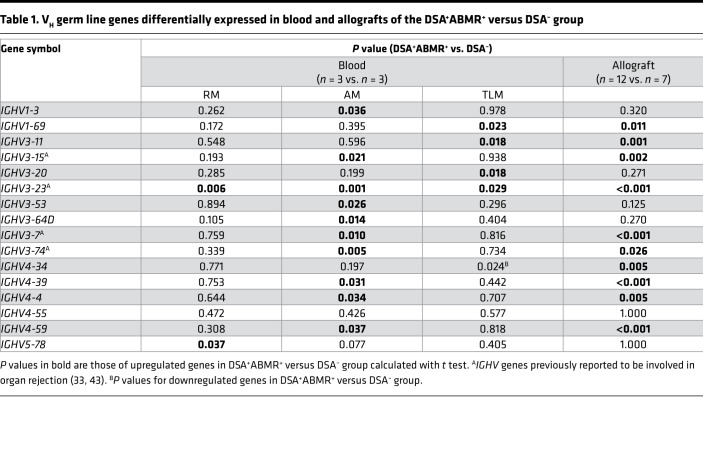
V_H_ germ line genes differentially expressed in blood and allografts of the DSA^+^ABMR^+^ versus DSA^–^ group

## Version 1 05/25/2021

In-Press Preview

## Version 2 06/22/2021

Electronic publication
