# Supplementary material for: T-bet+CD27+CD21– B cells poised for plasma cell differentiation during antibody-mediated rejection of kidney transplants
Source: JCI Insight. 2021 Jun 22;6(12):e148881. doi: 10.1172/jci.insight.148881 (PMC8262465; doi:10.1172/jci.insight.148881)
Supplement: Supplemental data [file jciinsight-6-148881-s090.pdf]

Supplemental data

Figure S1. Study outline and cross-sectional blood samples of kidney transplant patients

Figure S2. Flow cytometry analyses of blood B cells

Figure S3. SPADE cluster distribution across the patients' groups

Figure S4. Flow cytometry analyses of T-bet expression in blood B cell subsets

Figure S5. Flow cytometry analyses of MBCs subsets

Figure S6. Gating strategy for sorting naive B cells, MBCs and cT_{FH} cells

Figure S7. Transcriptional profiling of AM and TLM subsets in kidney transplant patients

Figure S8. Analysis of B cell phenotypes induced after B cell activation *in vitro*

Figure S9. Analysis of B cell phenotypes induced after B cell activation with different cytokines *in vitro*

Figure S10. Correlation of frequencies of MBC subsets with disease manifestations of ABMR

Figure S11. Dynamics of IgD, IL-21R and T-bet expression of AM subset, and correlation with timing to ABMR onset post-transplant

Figure S12. High-dimensional flow cytometry analyses of MBCs in individual patients

Figure S13. AM cells within kidney allografts of different patient groups

Figure S14. Molecular signatures of AM subsets within kidney allografts of patients

Table S1. Patients demographics (blood samples)

Table S2. Assay table and sample sizes

Table S3. Memory B cell clusters and phenotypic patterns

Table S4. GO pathways significantly upregulated in AM versus RM subset in DSA+ABMR+ group

Table S5. GO pathways significantly upregulated in TLM versus RM subset in DSA+ABMR+ group

Table S6. VH germ line genes differentially expressed in blood and allografts of DSA+ABMR- versus DSA- group

Table S7. Patients demographics (allograft biopsy samples)

Table S8. Antibodies for flow cytometry

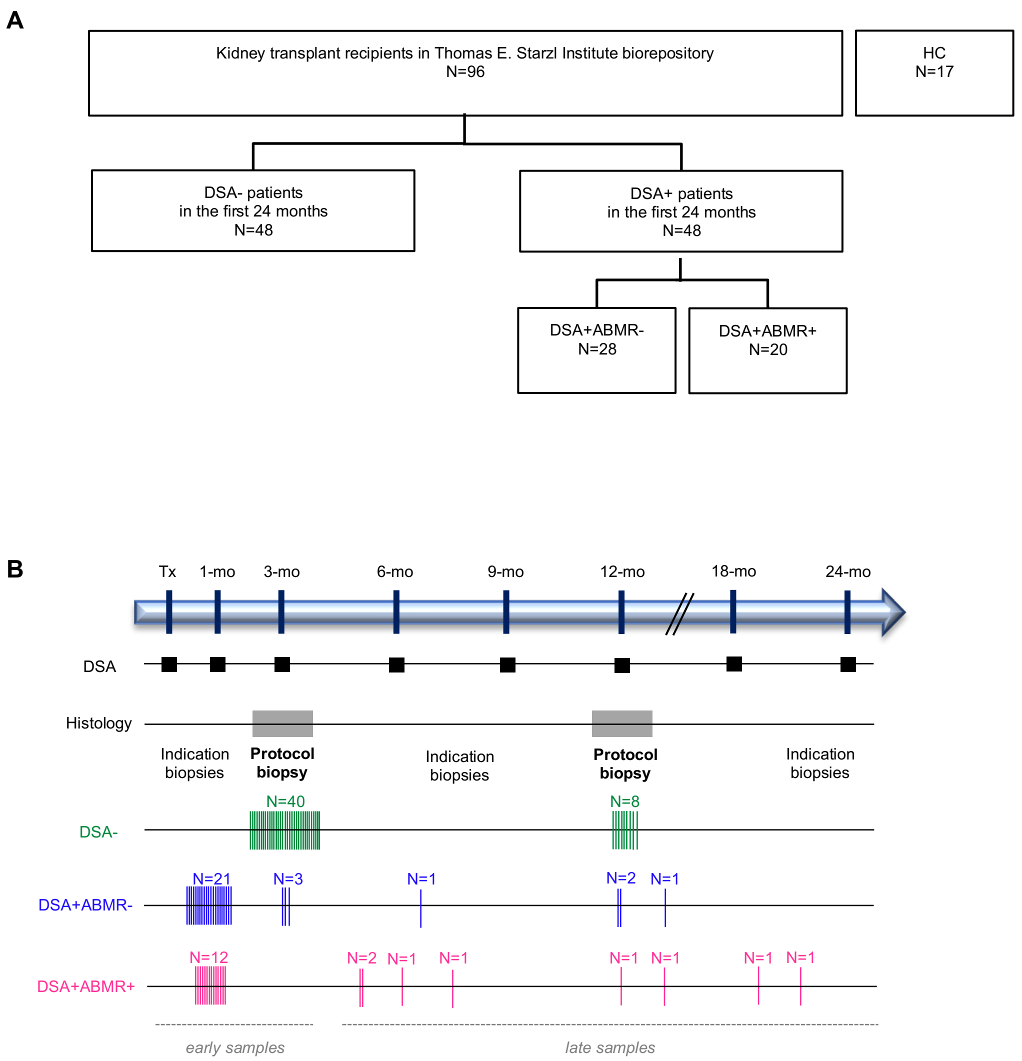

Figure S1. Study outline and cross-sectional blood samples of kidney transplant patients **(A)** Study design. Identification of three groups of patients according to the presence of post-transplant DSA and biopsy-proven ABMR status in the first 24 months post-transplant: patients without DSA nor ABMR (DSA-), patients with DSA without ABMR (DSA+ABMR-) and patients with DSA and ABMR (DSA+ABMR+). HC (healthy control) subjects served as control group. **(B)** Schematic representation of the screening strategy of patients for circulating DSAs; at 1-, 3-, 6-, 9-, 12-, 18- and 24-month post-transplant and at the time of indication biopsies. ABMR was detected by kidney allograft protocol and indication biopsies. Cross-sectional time points of blood samples are represented by vertical colored bars. Early samples are defined by samples collected ≤ 3-month and late samples are those collected >3-month post-transplant.

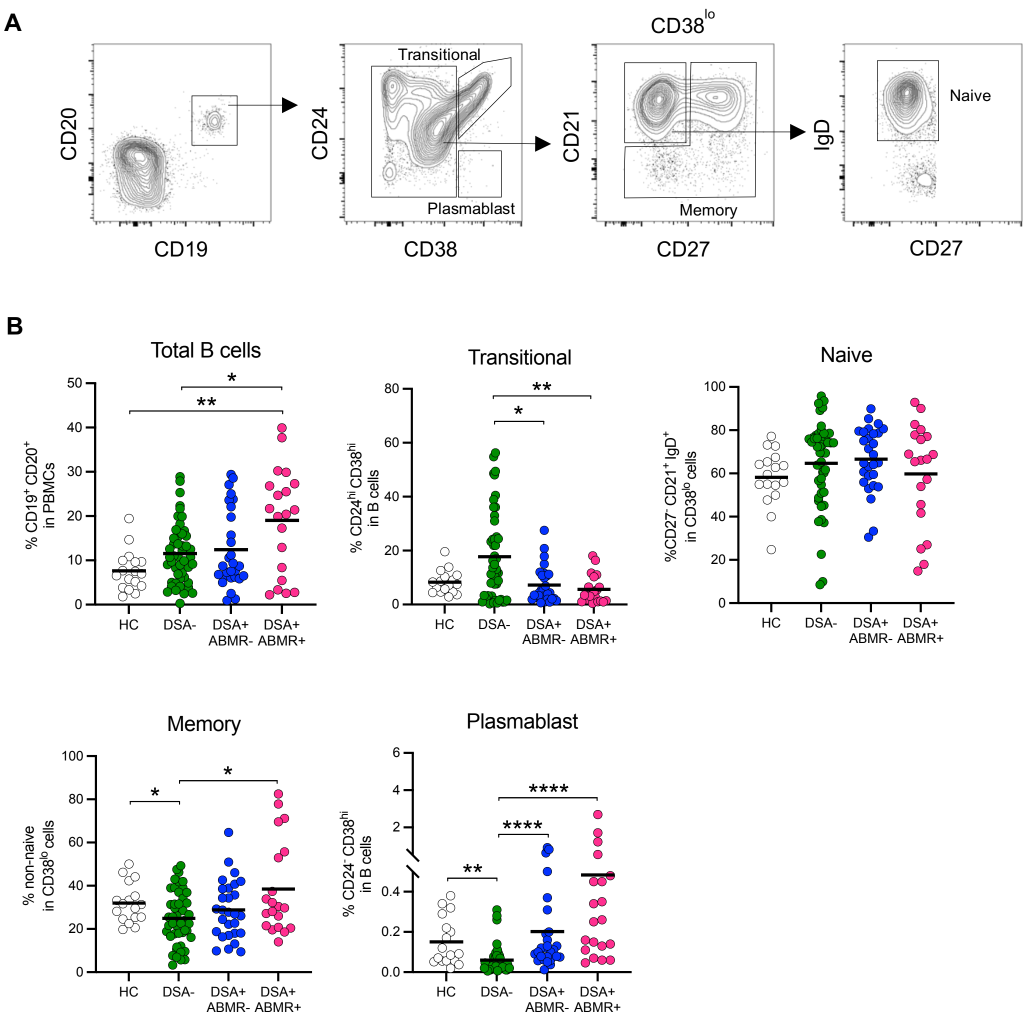

Figure S2. Flow cytometry analyses of blood B cells

(**A**) Representative example of the gating strategy by flow cytometry and (**B**) dot plots of percentages of total B cells ($CD19^{+}CD20^{+}$), transitional ($CD24^{hi}CD38^{hi}$), naive ($CD38^{lo}CD27^{-}CD21^{+}IgD^{+}$), MBCs (non-naive $CD38^{lo}$) and plasmablasts ($CD24^{-}CD38^{hi}$) are displayed; HC (N=17), DSA- (N=48), DSA+ABMR- (N=28) and DSA+ABMR+ (N=20) patients. Kruskal-Wallis with Dunn's post-test. *P < 0.05; **P < 0.01; ****P < 0.0001. Each dot represents one subject and horizontal lines are mean values.

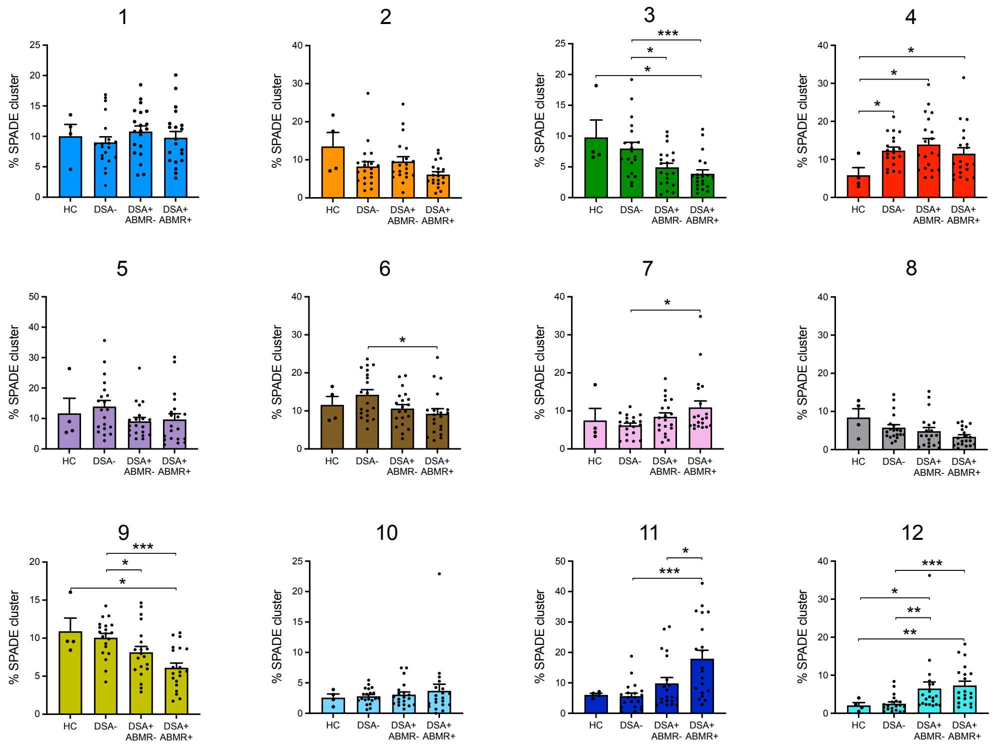

Figure S3. SPADE cluster distribution across the patients' groups

Bar plot showing MBC clusters' distribution based on SPADE clustering as in panel **1C**. Kruskal-Wallis with Dunn's post-test. *P < 0.05; **P < 0.01; ***P < 0.001. Each dot represents one subject and horizontal lines are mean values ± SEM.

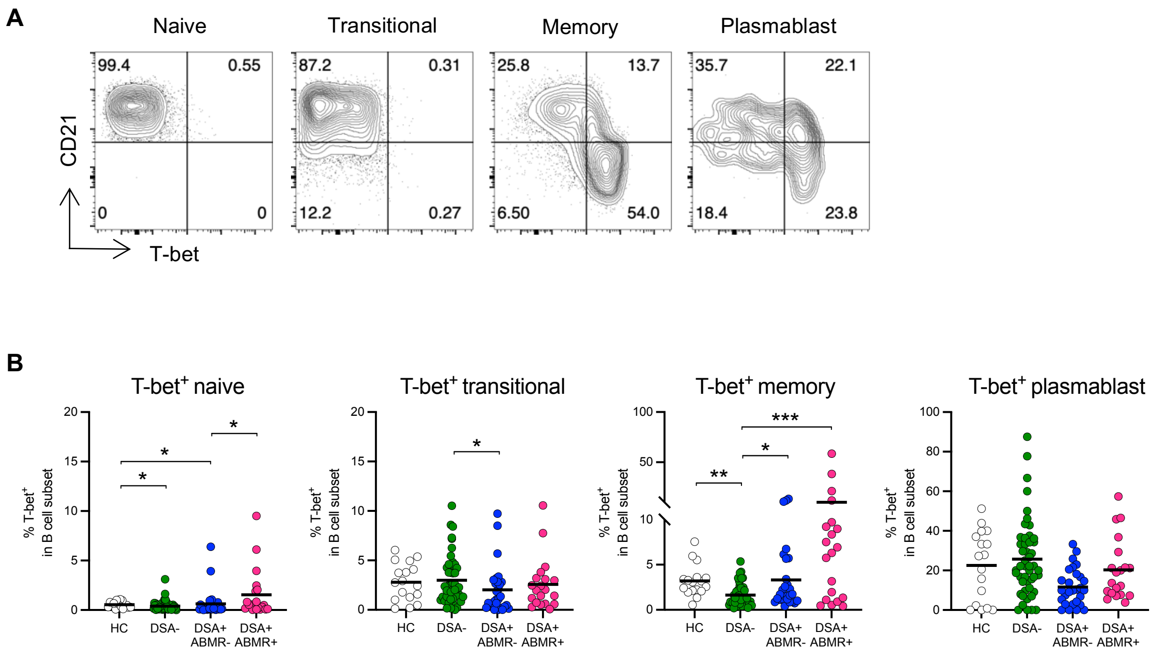

Figure S4. Flow cytometry analyses of T-bet expression in blood B cell subsets

(A) Representative examples of flow cytometry analysis and **(B)** dot plots of percentages of T-bet$^+$ cells in naive, transitional, MBCs and plasmablasts are displayed; HC (N=17), DSA- (N=48), DSA+ABMR- (N=28) and DSA+ABMR+ (N=20) patients. Kruskal-Wallis with Dunn's post-test. *P < 0.05; **P < 0.01; ***P < 0.001. Each dot represents one subject and horizontal lines are mean values.

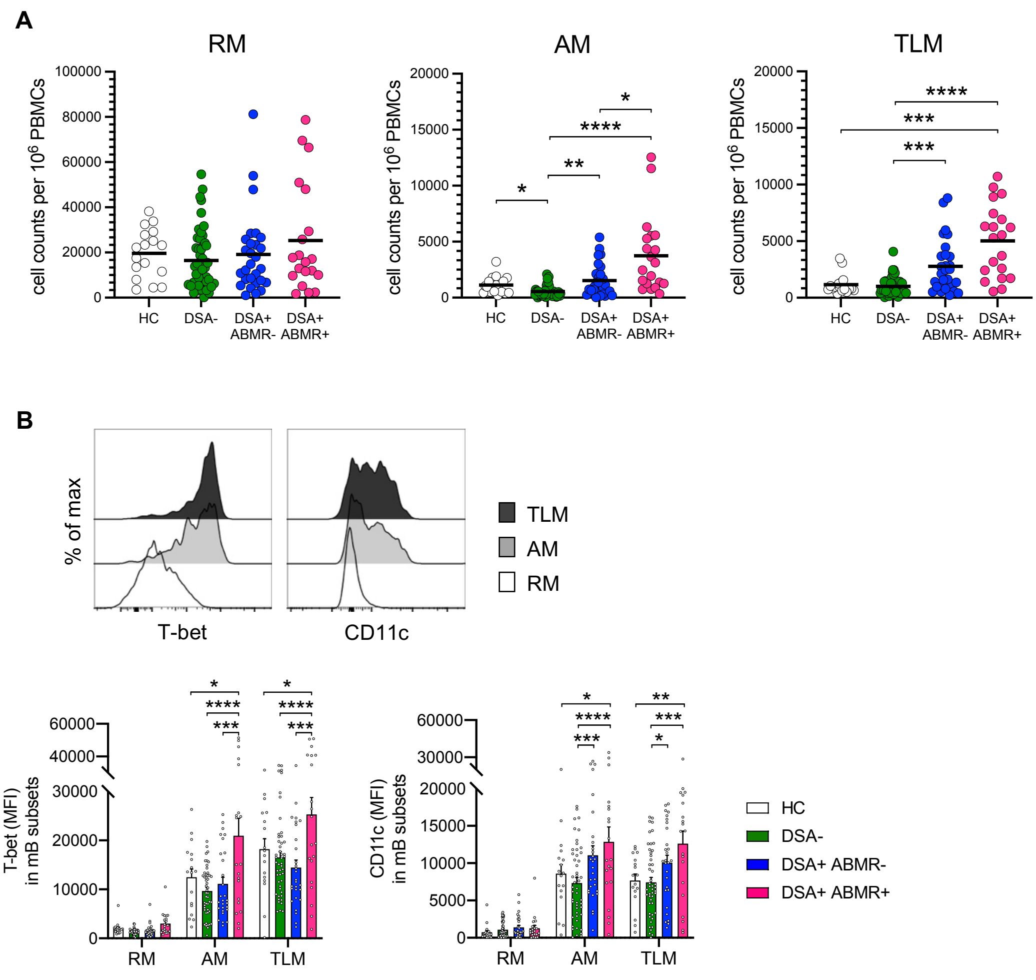

Figure S5. Flow cytometry analyses of MBCs subsets

(A) Dot plots of resting memory ($CD27^+CD21^+$, RM), activated memory ($CD27^+CD21^-$, AM) and tissue-like memory ($CD27^-CD21^-$, TLM) subsets, as cell counts per million PBMCs by flow cytometry are displayed; HC (N=17), DSA- (N=48), DSA+ABMR- (N=28) and DSA+ABMR+ (N=20) patients. **(B)** Representative examples of flow cytometry histograms and bar plots of MFI values of T-bet and CD11c in RM, AM and TLM subsets are displayed. Sample sizes as in panel **A**. Kruskal-Wallis with Dunn's post-test for panel **A**. Repeated measures two-way ANOVA with Tukey correction for panel **B**. *$P < 0.05$; **$P < 0.01$; ***$P < 0.001$; ****$P < 0.0001$. Each dot represents one subject and horizontal lines are mean values ± SEM.

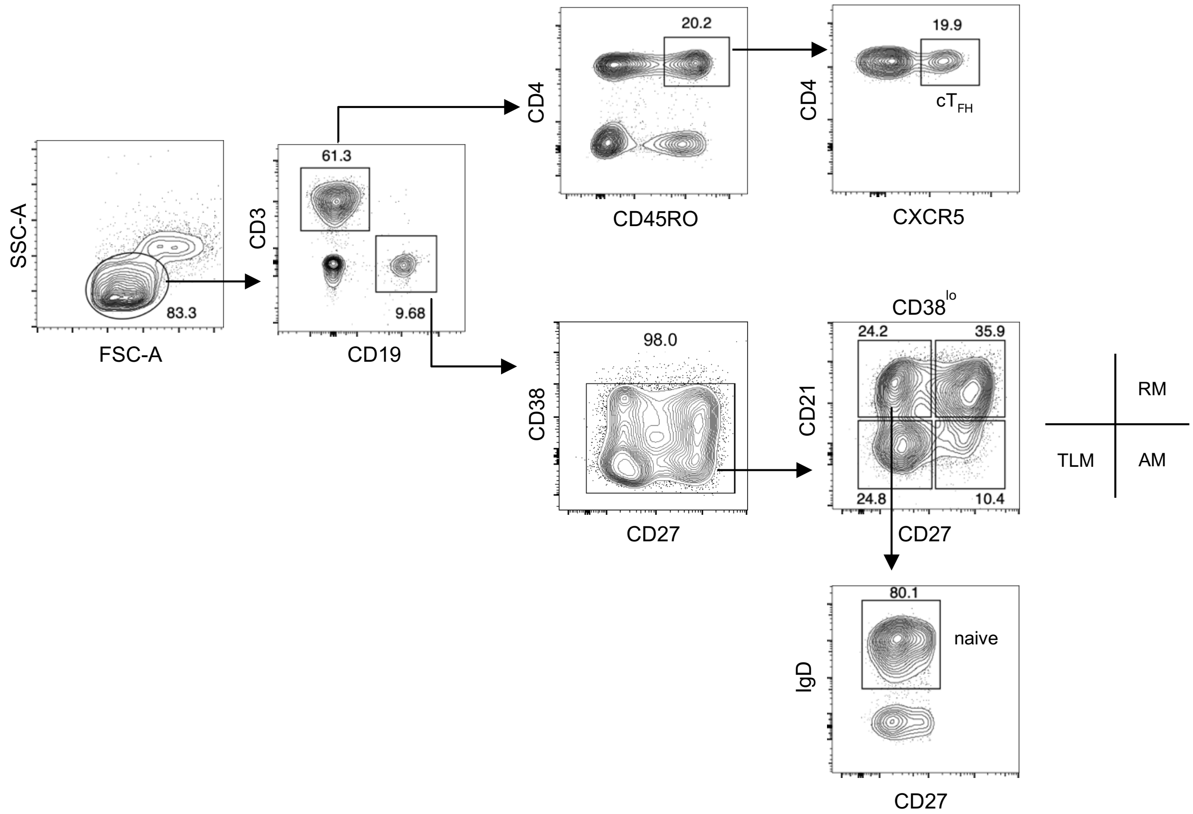

Figure S6. Gating strategy for sorting naive B cells, MBCs and cT$_{FH}$ cells

Representative example of the gating strategy for FACS sorting of naive (CD19$^+$CD3$^-$CD38^{lo}CD27$^-$CD21$^+$IgD$^+$) B cells, RM (CD19$^+$CD3$^-$CD38^{lo}CD27$^+$CD21$^+$), AM (CD19$^+$CD3$^-$CD38^{lo}CD27$^+$CD21$^-$), TLM (CD19$^+$CD3$^-$CD38^{lo}CD27$^-$CD21$^-$) and cT$_{FH}$ (CD19$^-$CD3$^+$CD4$^+$CD45RO$^+$CXCR5$^+$) cells from PBMC samples.

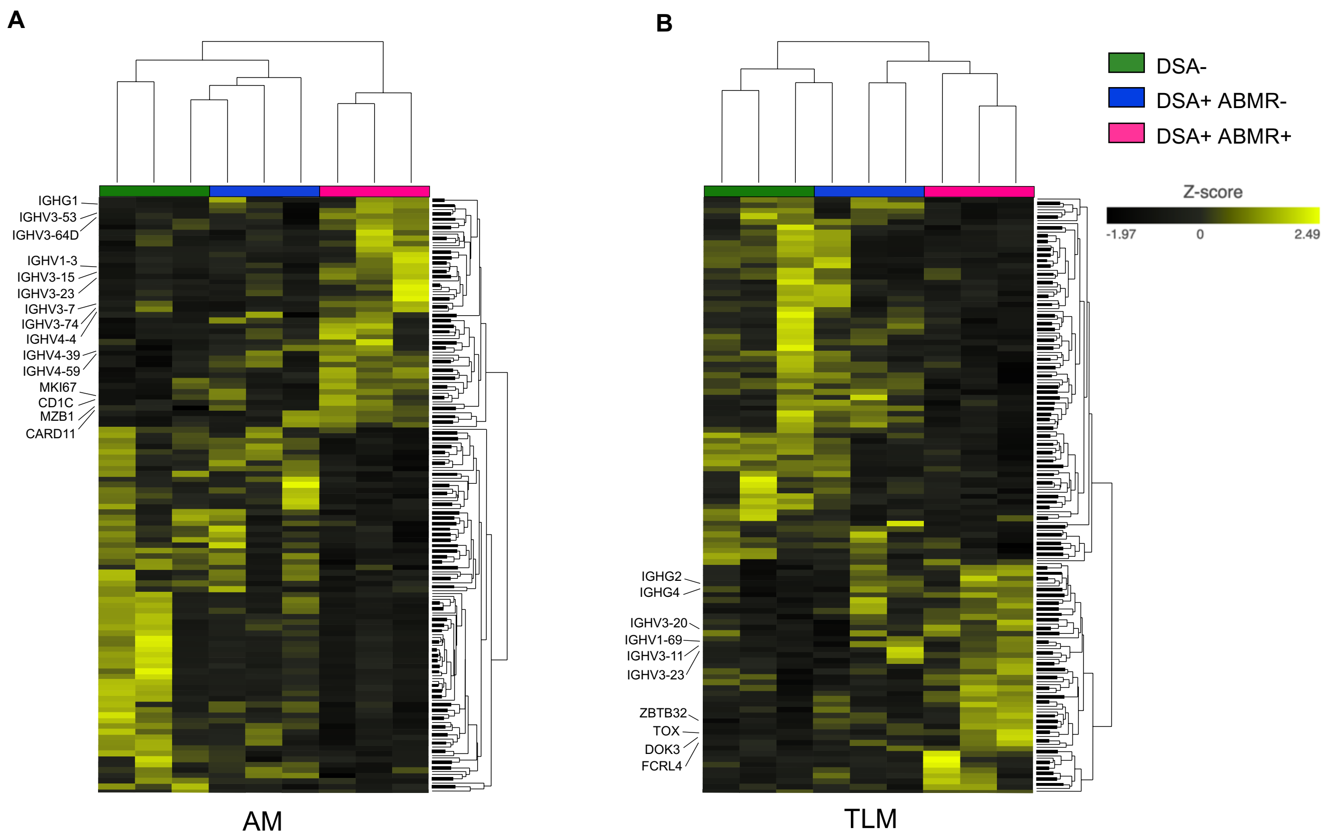

Figure S7. Transcriptional profiling of AM and TLM subsets in kidney transplant patients

RNA-seq analysis of AM and TLM subsets was performed in three patients per group; DSA- (N=3), DSA+ABMR- (N=3) and DSA+ABMR+ (N=3). Heatmaps generated by hierarchical clustering of genes and the three types of patient samples for AM **(A)** and TLM **(B)** subsets are displayed. Genes used for clustering were differentially expressed (fold change >1.5, false discovery rate P-Value <0.10).

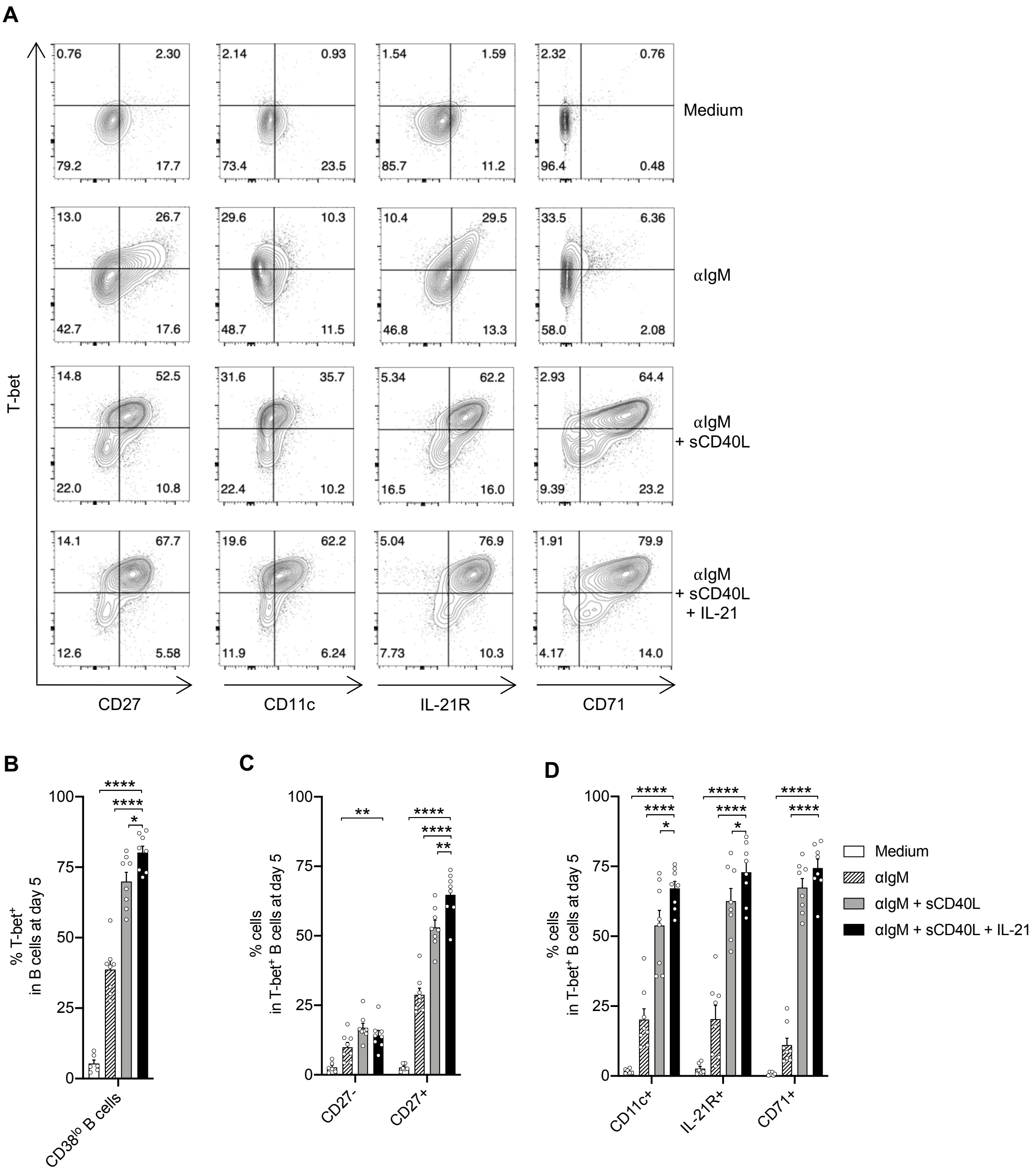

Figure S8. Analysis of B cell phenotypes induced after B cell activation *in vitro*

Naive ($CD19^{+}CD3^{-}CD38^{lo}CD27^{-}CD21^{+}IgD^{+}$) B cells were sorted from PBMCs of HC (N=8) and were activated with a combination of stimulators for 5 days. **(A)** Representative example of flow cytometry analysis of B cells after 5 days of culture after excluding $CD38^{hi}$ B cells. **(B)** Bar plots of percentages of T-bet^{+} cells in $CD38^{lo}$ B cells, **(C)** $CD27^{-}$ and $CD27^{+}$ cells in T-bet^{+} B cells, **(D)** CD11c^{+}, IL-21R^{+} and CD71^{+} cells in T-bet^{+} B cells after 5 days of culture, in each indicated stimulatory condition, are displayed. Multiple t-test with Holm-Sidak correction for panel **B**, **C** and **D**. *P < 0.05; **P < 0.01; ****P < 0.0001. Each dot represents one subject and horizontal lines of bars are mean values ± SEM.

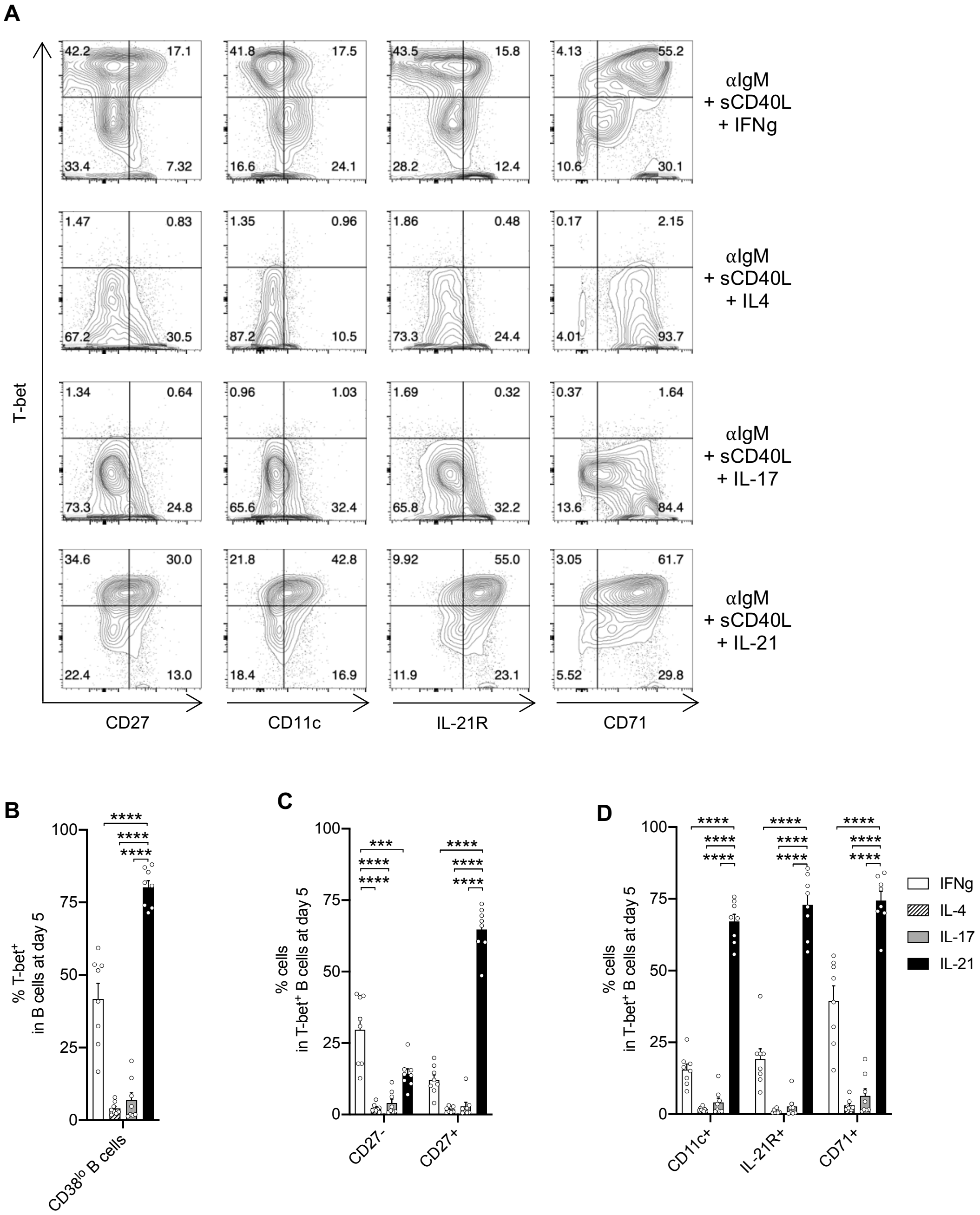

Figure S9. Analysis of B cell phenotypes induced after B cell activation with different cytokines *in vitro*

Naive ($CD19^+CD3^-CD38^{lo}CD27^-CD21^+IgD^+$) B cells were sorted from PBMCs of HC (N=8) and activated with a combination of cytokine cocktails for 5 days. **(A)** Representative example of flow cytometry analysis of B cells after 5 days of culture after excluding $CD38^{hi}$ B cells. **(B)** Bar plots of percentages of T-bet$^+$ cells in $CD38^{lo}$ B cells, **(C)** $CD27^-$ and $CD27^+$ cells in T-bet$^+$ B cells, **(D)** CD11c$^+$, IL-21R$^+$ and CD71$^+$ cells in T-bet$^+$ B cells after 5 days of culture, in each indicated stimulatory condition, are displayed. Multiple t-test with Holm-Sidak correction for panel **B**, **C** and **D**. ***$P < 0.001$; ****$P < 0.0001$. Each dot represents one subject and horizontal lines of bars are mean values ± SEM.

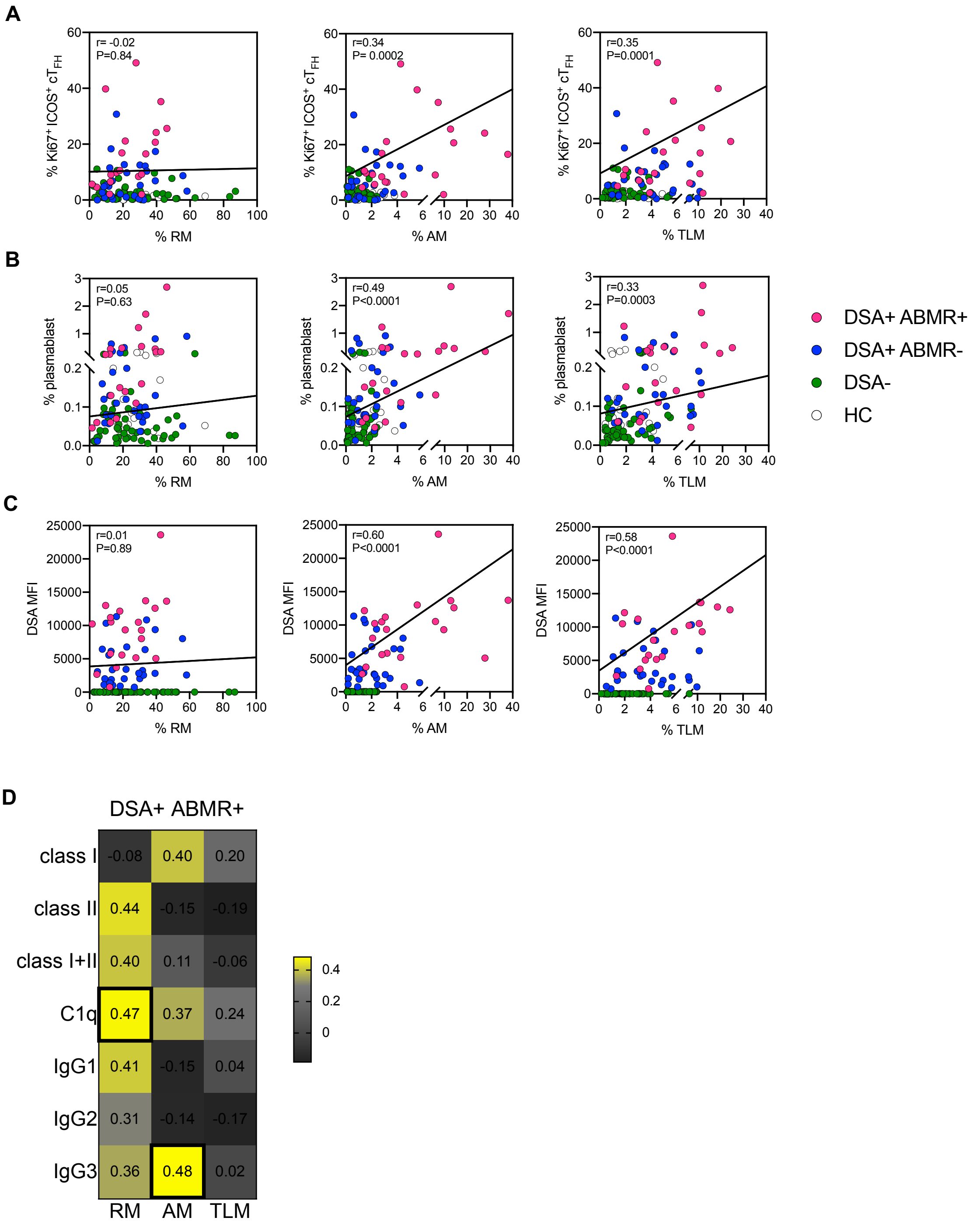

Figure S10. Correlation of frequencies of MBC subsets with disease manifestations of ABMR

Spearman correlation analysis of percentages of RM, AM and TLM subsets with percentages of blood **(A)** Ki67^{+}ICOS^{+} cT$_{FH}$ (CD3^{+}CD4^{+}CD45RO^{+}CXCR5^{+}) cells, **(B)** plasmablasts (CD19^{+}CD24^{-}CD38^{hi}) and **(C)** DSA MFI levels measured in serum by Luminex, are displayed; HC (N=17), DSA- (N=48), DSA+ABMR- (N=28) and DSA+ABMR+ (N=20). **(D)** Heatmap

showing Spearman correlation coefficients of percentages of RM, AM and TLM subsets with MFI levels of: class I, class II, sum of class I plus II, C1q-binding and IgG subclasses of DSAs measured in serum from DSA+ABMR+ patients, by Luminex. Bold squares indicate correlations with $P<0.05$. DSA class I and II analyses were performed for N=20, DSA IgG subclass analysis was performed for N=18 and DSA C1q-binding analysis was performed for N=19 patients.

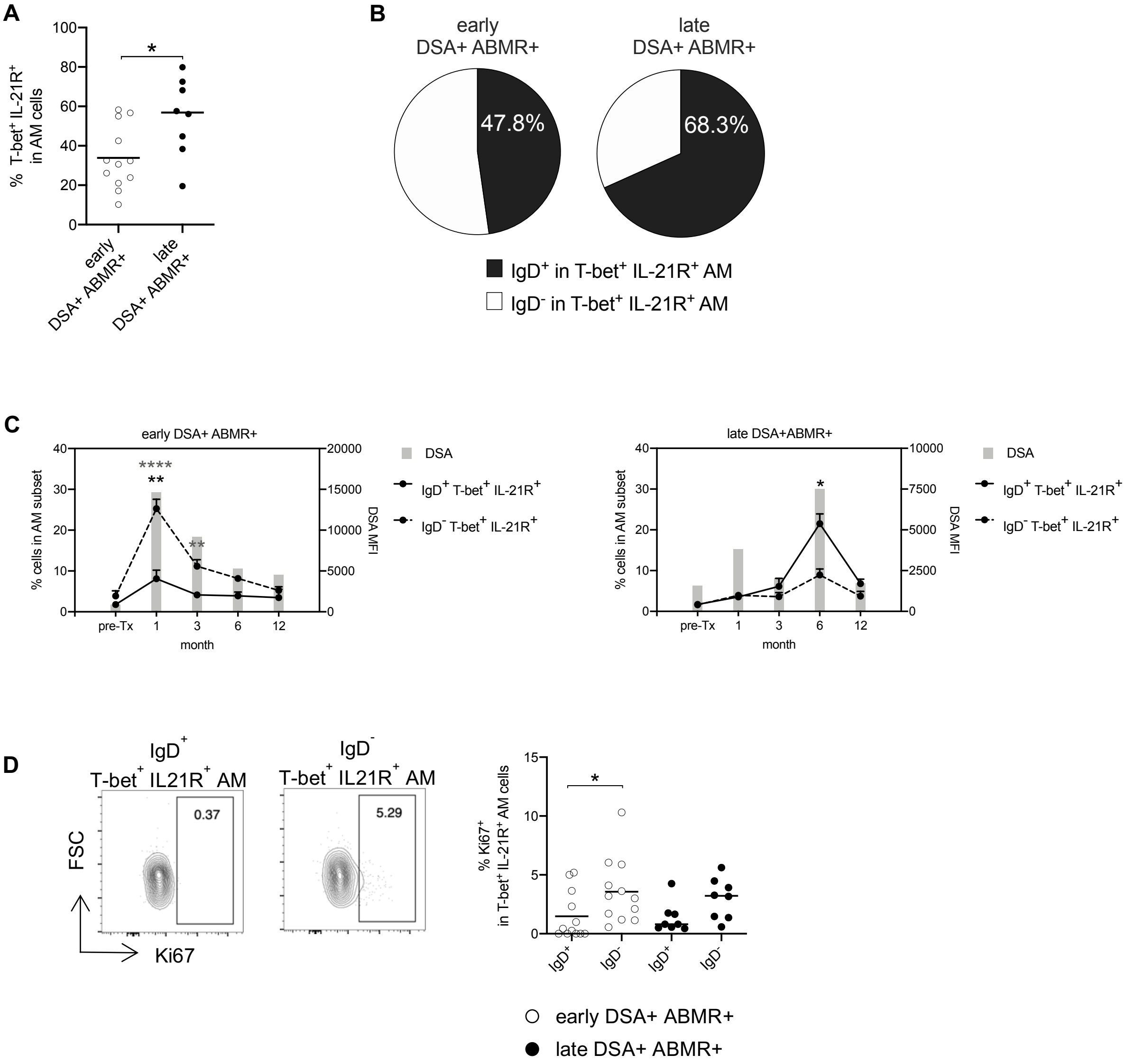

Figure S11. Dynamics of IgD, IL-21R and T-bet expression of AM subset, and correlation with timing to ABMR onset post-transplant

(A) Dot plot of percentages of T-bet$^+$ IL-21R$^+$ cells in AM cells, according to early (before 3-month) or late (after 3-month post-transplant) ABMR occurrence are displayed; early DSA+ABMR+ (N=12) and late DSA+ABMR+ (N=8) patients. **(B)** Pie charts of percentages of IgD$^+$ and IgD$^-$ cells in T-bet$^+$ IL-21R$^+$ AM cells are displayed; sample sizes as in panel **A**. **(C)** Patients were sampled longitudinally from pre-transplant to the indicated intervals in post-transplant. Kinetics of emergence of IgD$^+$ (bold connecting lines) and IgD$^-$ (dashed connecting lines) T-bet$^+$ IL-21R$^+$ in AM cells, and DSAs (grey bars) are displayed; early DSA+ABMR+ (N=3) and late DSA+ABMR+ (N=2) patients. Mixed-effects model for comparison of IgD$^+$ or IgD$^-$ T-bet$^+$ IL-21R$^+$ AM cells between time points. IgD$^+$ or IgD$^-$ T-bet$^+$ IL-21R$^+$ AM cell data

are shown as % ± SEM and DSA bars represent mean values. **(D)** Representative examples of flow cytometry analysis and dot plot of percentages of $Ki67^+$ in IgD^+ or IgD^- T-bet^+ IL-$21R^+$ AM cells, according to early or late ABMR occurrence are displayed; sample sizes as in panel **A**. Mann-Whitney U test for panel **A** and Wilcoxon matched-pairs signed rank test for panel **D**. *$P < 0.05$. Each dot represents one subject and horizontal lines are mean values.

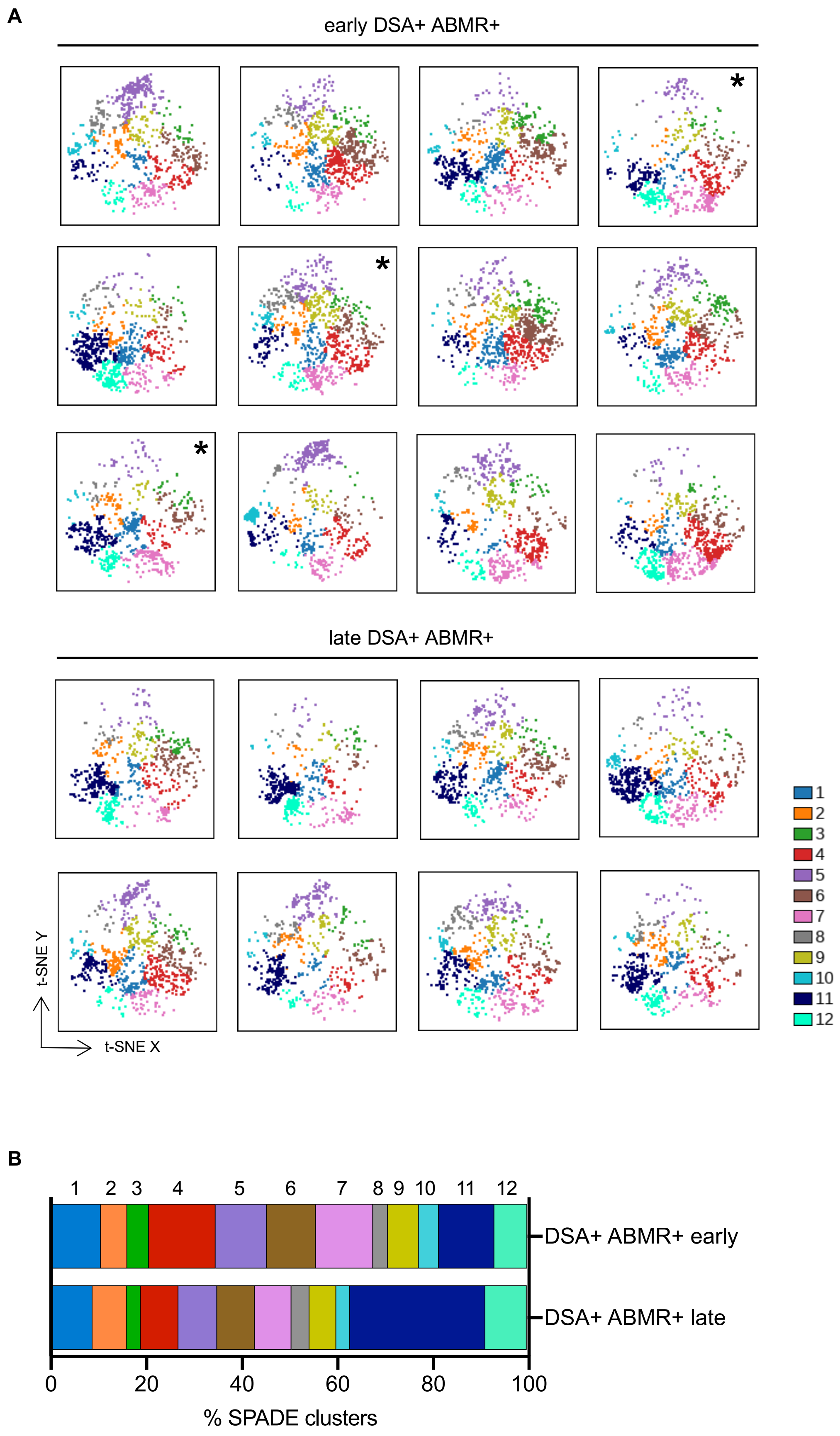

Figure S12. High-dimensional flow cytometry analyses of MBCs in individual patients

t-SNE projections of MBCs were overlaid with the 12 MBC clusters delineated by SPADE clustering (as in **Figure 1C**). **(A)** t-SNE maps were generated for individual patients from early DSA+ABMR+ (N=12) and late DSA+ABMR+ (N=8) groups. Each t-SNE map is based on N=990 cells. * indicates patients with pure DSA+ABMR+. **(B)** Stacked bar plot showing the 12 MBC clusters distribution based on SPADE clustering as in panel **A**. Clusters 4 and 11 are significantly different in their proportions across the indicated groups, by Mann-Whitney U test.

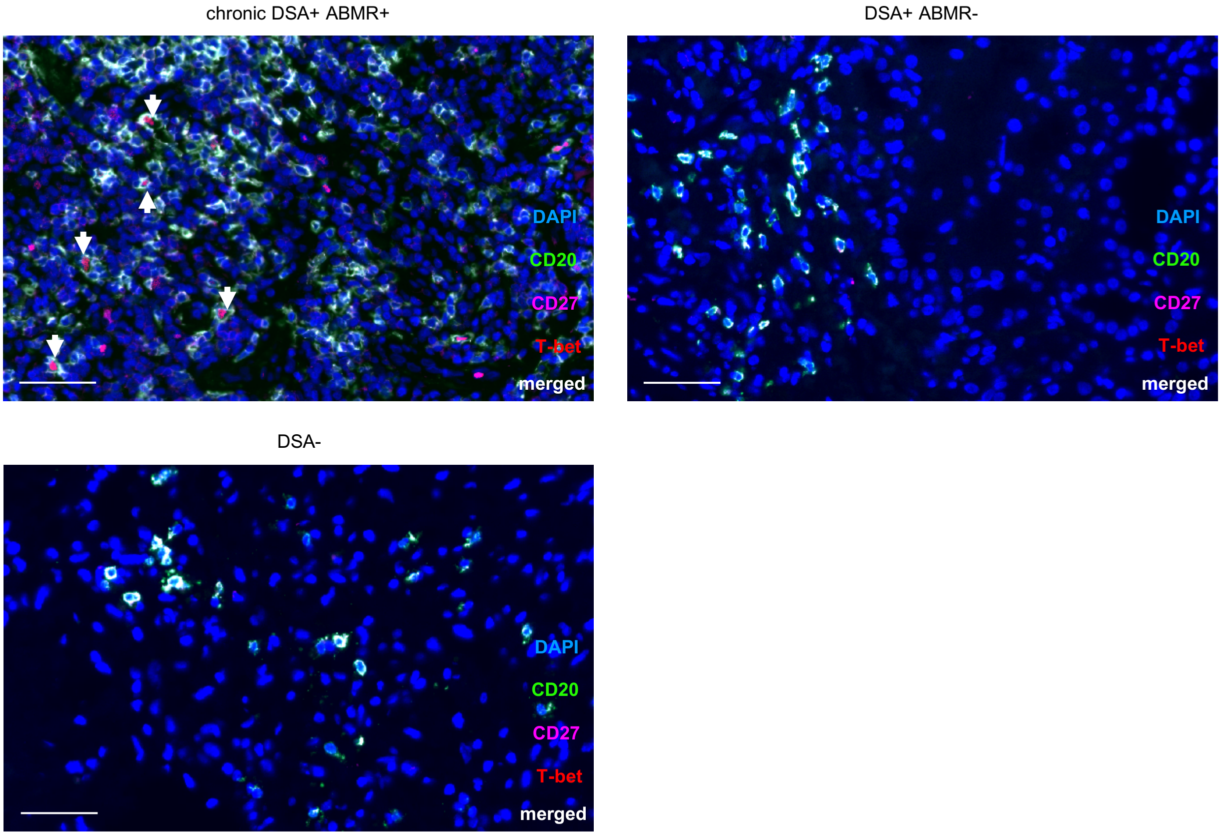

Figure S13. AM cells within kidney allografts of different patient groups

Representative multiplex immunofluorescence staining performed on kidney allograft biopsies from chronic DSA+ABMR+, DSA+ABMR- and DSA- patients. Each image is representative of two independent kidney allograft biopsy samples from each patient group. Stainings for chronic DSA+ABMR+ were performed on transplant explant biopsies from patients with incurable chronic DSA+ABMR+, refractory to immunosuppressive treatments. Arrows indicate $CD20^+$ $CD27^+$ T-bet^+ (triple-positive) cells. Scale bars indicate 50µm.

A

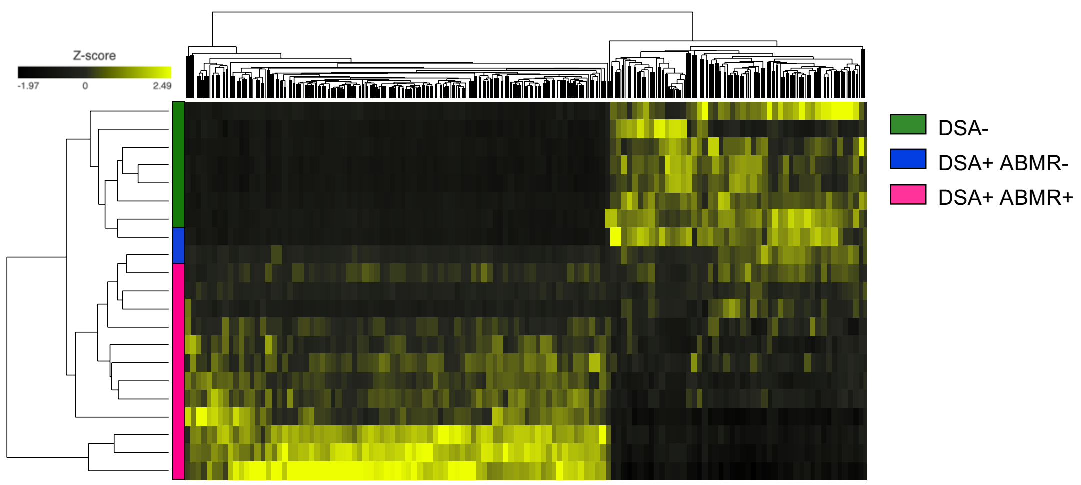

B

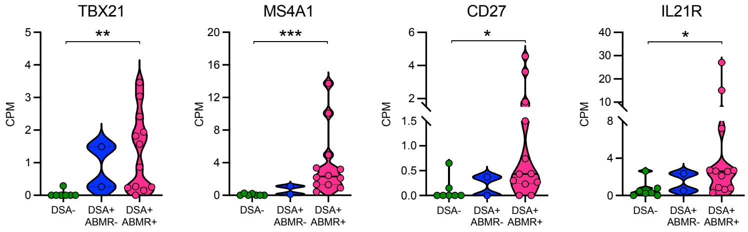

Figure S14. Molecular signatures of AM subsets within kidney allografts of patients

RNA-seq analysis of kidney allograft biopsies from 21 patients was performed; DSA- (N=7), DSA+ABMR- (N=2) and DSA+ABMR+ (N=12). **(A)** Heatmap generated by hierarchical clustering of genes and the three types of patient samples is displayed. Genes used for clustering were differentially expressed (fold change >2, false discovery rate P-Value <0.05). **(B)** Violin plots showing the expression levels of selected AM-specific genes from indicated patient groups. CPM, counts per million. Kruskal-Wallis with Dunn's post-test. *P < 0.05; **P < 0.01; ***P < 0.001. Each dot represents one subject and horizontal lines are median values ± SEM.

Table S1. Patients demographics (blood samples)

	HC	DSA-	DSA+ ABMR-	DSA+ ABMR+	P value****
	**N=17**	**N=48**	**N=28**	**N=20**	
**Characteristics at the time of transplantation**					
Recipient age (years), mean ± SD	50.9 ±13.1	49.8 ±13.7	47.3 ±14.3	45.3 ±15.1	0.5404
Recipient male sex, n(%)	6 (35.3)	33 (68.8)	16 (57.1)	11 (55.0)	0.1133
Caucasian, n(%)	16 (94.1)	43 (89.6)	26 (92.9)	15 (75.0)	0.1964
Retransplantation, n(%)	–	3 (6.3)	7 (25.0)	8 (40.0)	0.0031
Time in dialysis (months), mean ± SD	–	23.7 ± 29.8	43.6 ± 46.5	37.0 ± 28.8	0.0525
Native kidney disease, n(%)					
Glomerular*	–	9 (18.8)	11 (39.3)	5 (25.0)	0.1432
Hypertensive	–	6 (12.5)	2 (7.1)	4 (20.0)	0.4141
Tubulointerstitial nephropathy	–	1 (2.1)	3 (10.7)	1 (5.0)	0.2631
Polycystic kidney disease	–	8 (16.7)	5 (17.9)	2 (10.0)	0.7315
Diabetes	–	12 (25.0)	3 (10.7)	5 (25.0)	0.2932
Other nephropathy**	–	12 (25.0)	5 (17.9)	5 (25.0)	0.7509
Donor age (year), mean ± SD	–	43.5 ±12.3	39.1 ±15.3	39.4 ±13.7	0.3177
Donor male sex, n(%)	–	21 (43.8)	16 (57.1)	10 (50.0)	0.5272
Living donor, n(%)	–	25 (52.1)	11 (39.3)	5 (25.0)	0.1096
Cold ischemia time (min), mean ± SD	–	310.1 ± 368.8	465.3 ± 398.0	417.5 ± 299.3	0.1795
Thymoglobulin induction therapy, n(%)	–	48 (100)	28 (100)	20 (100)	–
Negative flow cytometry crossmatch, n(%)	–	48 (100)	28 (100)	20 (100)	–
HLA mismatches (number), mean ± SD***	–	5.0 ± 2.0	5.3 ± 1.9	5.0 ± 2.1	0.8571
DSA present before transplantation, n(%)	–	0 (0)	11 (39.3)	12 (60.0)	<0.0001
**Characteristics at the time of cross-sectional sample collection**					
Time to transplantation (months), mean ± SEM	–	4.5 ± 0.4	3.8 ± 0.7	5.3 ± 1.4	0.4668
Tacrolimus, n(%)	–	48 (100)	27 (96.4)	18 (90.0)	0.0959
Tacrolimus trough level (µg/L), mean ± SD	–	9.5 ± 3.1	9.3 ± 2.9	8.8 ± 5.3	0.7336
Mycophenolate mofetil, n(%)	–	48 (100)	28 (100)	20 (100)	–

ABMR, antibody-mediated rejection ; DSA, donor-specific antibody ; HLA, human leukocyte antigen ; TCMR, T-cell mediated rejection

*Glomerulopathies includes chronic glomerulonephritis, crescentic glomerulonephritis, focal segmental glomerulosclerosis, Wegener's granulomatosis, IgA nephropathy, membranous nephropathy

**other nephropathy includes familial nephropathy, renal hypoplasia and other noncategorized conditions

***HLA mismatches at A, B, DR and DQ locus

****One-way ANOVA and chi-squared test were used for statistical comparison of continuous and categorical variables, respectively

Table S2. Assay table and blood sample sizes

	HC	DSA-	DSA+ ABMR-	DSA+ ABMR+	total
**PBMC profiling**					
B cell 21-color flow cytometry	17	48	28	20	113
T cell 22-color flow cytometry	17	48	28	20	113
MBC RNA-seq	–	3	3	3	9
T_{FH}/MBC co-cultures	4	3	3	7	17
B cell activation *in vitro*	8	–	–	–	8
**Serum profiling**					
DSA MFI, class, specificity	–	–	28	20	48
DSA C1q-binding	–	–	23	19	42
DSA IgG subclasses	–	–	18	18	36

MBC, memory B cells; T_{FH}, T follicular helper cells; MFI, mean fluorescence intensity

Table S3. Memory B cell clusters and phenotypic patterns

cluster	markers	IL-21R expression	isotype switching	proliferation	mB subset
**1**	CD21+ CD24+ CD27+ CXCR5+ IgD+	No	unswitched	No	RM
**2**	CD21+ CD24+ CXCR5+ IgD+ CD27+/-	No	unswitched	No	RM
**3**	CD21+ CD24+ CD38+ CXCR5+ IgD+ CD27+ CD86+	No	unswitched	No	RM
**4**	CD21+/- CD24+ CD38+ CXCR5+ IgD- CD27+ CD86+	No	switched	No	AM
**5**	CD21+ IL21R+ CD24+ CD38+ CXCR5+ IgD+ CD27- CD86+ CD95+ Ki67+	Yes	unswitched	Yes	RM
**6**	CD21+ CD24+ CD38+ CXCR5+ IgD- CD27+/-	No	switched	No	RM
**7**	T-bet+ CD11c+ CD21+/- CD24- CD38+ CXCR5+ IgD- CD27+ CD86+ Ki67+	No	switched	Yes	AM
**8**	CD21+ CD24+ CD38+ CXCR5+ IgD+ CD27- CD86+	No	unswitched	No	RM
**9**	CD21+ CD24+ CD38+ CXCR5+ IgD+ CD27-	No	unswitched	No	RM
**10**	CD21+ CD24+/- CD38+ CXCR5+ IgD+ CD27- IL6R+	No	unswitched	No	RM
**11**	T-bet+ CD11c+ CD19+ CD20+ CD21- IL21R+ IgD+ CD27+/- CD86+ Ki67+	Yes	unswitched	Yes	AM, TLM
**12**	T-bet+ CD11c+ CD19+ CD20+ CD21- IL21R+ IgD- CD27+/- CD86+ CD95+	Yes	switched	No	AM, TLM

Isotype switching was defined according to expression of IgD

Proliferation was defined according to expression of Ki67

Resting (RM), activated (AM) and tissue-like memory (TLM) were identified according to expression of CD21, CD27 and T-bet

Table S4. GO pathways significantly upregulated in AM versus RM subset in DSA+ABMR+ group

GO ID	GO Term	-log (Pvalue)	genes
GO:0006898	receptor-mediated endocytosis	9.25	MARCO, SCARF1, APOB, CTTN, TF, VEGFA, SPARC, PIKFYVE, AAK1, CLU, SAG, JCHAIN, SYT11, CD36, DAB2, ALB, SYK, HSP90B1, CXCL8, LDLRAD3, CXCR2, ITSN2, MIR27A, IGKV3D-20, IGLV1-51, IGLV2-23, IGHV3-7, IGHV3-53, IGKV2D-30, IGKV1D-33, IGKV1-17, IGKV2-28, MRC1, IGHV2-70
GO:0051256	mitotic spindle midzone assembly	4.39	KIF4A, KIF23, AURKB, PRC1
GO:0007094	mitotic spindle assembly checkpoint	4.33	MAD1L1, TPR, CENPF, TEX14, BUB1B, GEN1, AURKB
GO:0000281	mitotic cytokinesis	4.23	ANLN, KIF4A, ECT2, ESPL1, NUSAP1, KIF23, KIF20B, JTB, ANK3, PRC1
GO:0051301	cell division	4.07	MAD1L1, ANLN, TPR, ASPM, SPAG5, TPX2, KIF4A, KIF3B, VEGFA, ECT2, CENPF, ESRRB, TEX14, ZWINT, E2F8, ESPL1, NUSAP1, KIF23, KNL1, CUZD1, KIF20B, FGF2, CENPE, LEF1, KIF2C, JTB, CDCA5, ANK3, BUB1B, CDC25C, SPC24, SPICE1, CDC25A, ANAPC10, CDK5, E2F7, CCNE2, AURKB, MACC1, KIF18B, PDGFA, PRC1, SPOUT1, SPIRE2, KIFC1
GO:0050900	leukocyte migration	4.03	CD84, APOB, DDT, VEGFA, PIKFYVE, FN1, IL1R1, CSF3R, SDC4, GPR18, JCHAIN, ITGAX, S100A8, PLA2G7, JAML, NLRP3, S100A9, F2RL1, SYK, CXCL8, OLR1, UMODL1, CXCR2, DPP4, IGKV3D-20, IGLV1-51, IGLV2-23, IGHV3-7, IGHV3-53, IGKV2D-30, IGKV1D-33, IGKV1-17, IGKV2-28, IGHV2-70
GO:0016446	somatic hypermutation of immunoglobulin genes	3.44	POLQ, SAMHD1, AICDA, EXO1
GO:0002377	immunoglobulin production	3.39	POLQ, SAMHD1, AICDA, FGL2, FCRL3, IL7R, EXO1, CD28, IGKV3D-20, IGKV3D-11, IGLV5-52, IGLV1-51, IGLV2-23, IGKV3D-7, IGKV2D-30, IGKV1D-33, IGKV1-17, IGKV2-28, IGKV1-27
GO:0050853	B cell receptor signaling pathway	3.30	ITK, FOXP1, GPS2, FCRL3, MNDA, CTLA4, SYK, IGHV3-7, IGHV3-20, IGHV3-53, IGHV3-66, IGHV2-70

Table S5. GO pathways significantly upregulated in TLM versus RM subset in DSA+ABMR+ group

GO ID	GO Term	-log (Pvalue)	genes
GO:0007155	cell adhesion	5.53	BAIAP2L1, FARP2, TENM1, SLAMF7, VCAN, CD84, TRO, CBFB, ADGRL1, FCGR2B, TBX21, SCARF1, KIFAP3, GPC4, ACTN2, CTTN, CASS4, NRCAM, SEMA6A, JAK2, GRAP2, HCK, JAG1, PTPRS, SIGLEC6, HSPB1, CNTNAP3, MAP3K8, CRTAM, FOLR3, TRPV4, CLEC4A, SMOC2, BCL6, HHLA2, ADAM23, ZAP70, SLAMF1, KIF14, NRP2, CTGF, CTNNAL1, NR4A3, CSF3R, ADGRE5, CDH26, SOX4, WNT1, MACF1, PALLD, THEMIS2, LILRB2, VSTM2L, VAV3, PTPN22, DTX1, CD36, CD164, PLXNC1, THSD1, CD72, LRRC32, THBS1, ENTPD1, LEF1, ITGAX, SKAP1, S100A8, RHOB, HSPD1, BOC, LPP, TNFRSF21, PARD3, FEZ1, ANK3, MAGI, PTPRO, DST, UBASH3B, JAM2, PCDH1, ITGAD, SPON2, ITGB2, DISC1, FBLN2, CD200R1, SYK, INPPL1, SERPINB8, IL12A, EFNA1, PTK2, CXCL, LIMS1, MTSS1, FPR2, RASGRP1, PTPRM, SUSD5, PTPN2, ZNF645, CDK5R1, EPHB3, CADM1, ZNF703, PCDH9, GP1BA, GCNT1, NTNG2, DPP4, PDCD1LG2, DLL1, PPP1CB, PLXNA4, PCDHGC3, PCDHGC4, PCDHGA11, PCDHGB1, PCDHGA4, SRGAP2
GO:0006897	endocytosis	3.88	FGR, HEATR5B, PIK3C2A, SLC11A1, NCKAP1, SYT1, RAB27A, FCGR2B, SCARF1, DGKD, CTTN, SNX5, GRK3, RIN3, HCK, EEA1, EHD4, CLIP3, CEACAM4, SPARC, PIKFYVE, AAK1, SLAMF1, ESYT2, RHOQ, CLU, TMEM175, HEATR5A, SNX9, SAG, SYT11, VAV3, CD36, SDCBP, TMPRSS13, THBS1, BMP2K, MTMR6, STON2, FCGR2A, ACKR2, LRRTM2, RABGAP1L, DAB2, RABGEF1, SPON2, ITGB2, CD3G, NOSTRIN, SYK, INPPL1, LRP1B, PTK2, CXCL8, REPS2, FPR2, MCTP1, SNX18, LDLRAD3, APOBR, CLN3, LRRK2, MYO6, ITSN2, DLL1, INPP5F, IGLV6-57, IGHD, IGHV3-11, IGHV3-20, IGHV2-26, TXNDC5, IGKV2D-30, IGKV1D-33, CD302, STON1, IGLL5, XKR, IGHV2-70, IGHV4-4
GO:0032466	negative regulation of cytokinesis	3.76	TEX14, E2F8, E2F7, AURKB
GO:0043087	regulation of GTPase activity	3.67	RTN4R, RAP1GAP, GPR137B, RAPGEF3, RGS1, RIN3, ARHGEF10, ADAP1, BCL6, ECT2, RGS2, MKKS, DOCK4, CHN1, SNX9, VAV3, PLXNC1, SPRY2, BCAR3, FGD4, GPR65, IQGAP2, ARHGAP18, RGS18, RABGAP1L, RASGRP3, RANBP2 AGAP1, RAPGEF6, GPSM1, PTK2, LIMS1, RASGRP1, NET1, SNX18, EPHB3, RGPD6, IQGAP3, ADAP2, LRRK2, SRGAP1, SIPA1L1, ARHGAP11A, PLXNA4, ARFGAP3, SRGAP2
GO:0050855	regulation of B cell receptor signaling pathway	3.42	PRKCH, CBFB, FCGR2B, PTPN22, CMTM3, FCRL3, CD19
GO:0050919	negative chemotaxis	3.35	SEMA6A, NRP2, NRG1, SEMA4C, SLIT1, SEMA4A, PDGFA, DPP4, PLXNA4
GO:0000122	negative regulation of transcription by RNA polymerase II	3.18	ZBTB32, TPR, PER3, PRDM1, CBFB, MEF2A, TP63, TBX21, FGFR1, RBL1, ATRX, DNMT3B, NFATC2, TFEC, AEBP1, KLF3, TRPV4, BCL6, DLX2, NR4A3, DNMT3A, MXI1, BATF3, ATXN1, SIX1, SMO, E2F8, ZMYM5, MEIS2, CD36, PRDM5, LEF1, PRDM16, HMBOX1, NR6A1, WWC2, DAB2, NR4A2, JAZF1, BACH1, IRX6, TAL1, GFI1, DACT1, E2F7, ZNF608, EFNA1, ZEB2, WNT10B, CHD3, BPTF, PARP15, HOXB2, PTPN2, MAF, AURKB, HES7, MITF, PEG3, ZNF254, OTUD7B, UHRF1

GO:0033262	regulation of nuclear cell cycle DNA replication	2.99	FGFR1, ATRX, AICDA, BCL6, TERF1
GO:0000083	regulation of transcription involved in G1/S transition of mitotic cell cycle	2.97	CDK14, ORC1, CDC6, BACH1, GFI1, E2F7, RRM2

Table S6. V_H germ line genes differentially expressed in blood and allografts of DSA+ABMR- versus DSA- group

Gene Symbol	P Value (DSA+ ABMR- vs DSA-)			
	Blood (N=3 vs N=3)			Allograft (N=2 vs N=7)
	RM	AM	TLM	
IGHV1-3	0.703	0.908	0.352	1.000
IGHV1-69	0.281	1.000	0.201	1.000
IGHV3-11	0.077	0.818	0.288	1.000
IGHV3-15*	0.725	0.543	0.999	1.000
IGHV3-20	0.814	0.275	0.400	1.000
IGHV3-23*	0.393	0.409	0.573	1.000
IGHV3-53	0.372	**0.037**	0.667	1.000
IGHV3-64D	0.621	0.173	0.706	1.000
IGHV3-7*	0.773	0.189	0.688	0.444
IGHV3-74*	0.923	0.257	0.203	1.000
IGHV4-34	0.763	**0.007**	0.005[#]	1.000
IGHV4-39	0.293	0.471	0.339	1.000
IGHV4-4	0.543	0.902	0.157	1.000
IGHV4-55	**0.034**	0.822	0.705	1.000
IGHV4-59	0.773	0.964	0.120	0.444
IGHV5-78	0.168	0.692	0.033[#]	1.000

P values in bold are those of significantly upregulated genes in DSA+ABMR- versus DSA- group

* indicates IGHV genes previously reported to be involved in organ rejection[33, 34]

[#] indicates P values for significantly downregulated genes in DSA+ABMR- versus DSA- group

Table S7. Patients demographics (allograft biopsy samples)

	DSA-	DSA+ ABMR-	DSA+ ABMR+	P value****
	N=7	N=2	N=12	
**Characteristics at the time of transplantation**				
Recipient age (years), mean ± SD	50.6 ±11.3	53.5 ± 3.5	42.8 ± 12.8	0.2867
Recipient male sex, n(%)	5 (71.4)	1 (50.0)	6 (60.0)	0.646
Caucasian, n(%)	4 (57.1)	2 (100)	10 (83.3)	0.307
Retransplantation, n(%)	2 (28.6)	1 (50.0)	3 (25.0)	0.769
Time in dialysis (months), mean ± SD	8.1 ± 19.0	20.5 ± 16.3	53.2 ± 43.2	0.045
Native kidney disease, n(%)				
Glomerular*	0 (0)	1 (50.0)	5 (41.7)	0.119
Hypertensive	1 (14.3)	0 (0)	2 (16.7)	0.823
Tubulointerstitial nephropathy	0 (0)	0 (0)	0 (0)	–
Polycystic kidney disease	0 (0)	1 (50)	0 (0)	0.007
Diabetes	0 (0)	0 (0)	1 (8.3)	0.675
Other nephropathy**	6 (85.7)	0 (0)	4 (33.3)	0.032
Donor age (year), mean ± SD	48.2 ± 15.3	37.0 ± 5.7	44.4 ± 9.1	0.476
Donor male sex, n(%)	2 (28.6)	1 (50.0)	6 (50.0)	0.646
Living donor, n(%)	5 (71.4)	1 (50.0)	2 (16.7)	0.056
Cold ischemia time (min), mean ± SD	358.0 ± 514.5	331.0 ± 425.7	407.8 ± 203.6	0.933
Thymoglobulin induction therapy, n(%)	7 (100)	2 (100)	12 (100)	–
Negative flow cytometry crossmatch, n(%)	7 (100)	2 (100)	12 (100)	–
HLA mismatches (number), mean ± SD***	4.3 ± 2.9	5.0 ± 1.4	4.8 ± 1.7	0.848
**Characteristics at the time of cross-sectional sample collection**				
Time to transplantation (months), mean ± SEM	5.4 ± 1.7	7.6 ± 4.5	14.3 ± 4.5	0.338
TCMR lesions at the time of sample collection, n(%)	0 (0)	0 (0)	11 (91.6)	0.0002
Tacrolimus, n(%)	7 (100)	2 (100)	11 (91.7)	0.675
Tacrolimus trough level (µg/L), mean ± SD	8.2 ± 2.6	11.4 ± 2.5	8.9 ± 4.2	0.565
Mycophenolate mofetil, n(%)	7 (100)	2 (100)	11 (91.7)	0.675

ABMR, antibody-mediated rejection ; DSA, donor-specific antibody ; HLA, human leukocyte antigen ; TCMR, T-cell mediated rejection

*Glomerulopathies includes chronic glomerulonephritis, crescentic glomerulonephritis, focal segmental glomerulosclerosis, Wegener's granulomatosis, IgA nephropathy, membranous nephropathy

**other nephropathy includes familial nephropathy, renal hypoplasia and other noncategorized conditions

***HLA mismatches at A, B, DR and DQ locus

****One-way ANOVA and chi-squared test were used for statistical comparison of continuous and categorical variables, respectively

Table S8. Antibodies for flow cytometry

Marker	Dye	Clone	Source
Blimp1	AF647	6D3	BD Biosciences
CD11c	BV510	B-ly6	BD Biosciences
CD19	APC-eFluor 780	SJ25C1	Invitrogen
CD20	BV570	2H7	Biolegend
CD21	APC	Bu32	Biolegend
CD24	BV605	ML5	BD Biosciences
CD27	PE-Cy7	O323	Invitrogen
CD3	BV750	SK7	Biolegend
CD3	BV510	SK7	Biolegend
CD32	BV605	FLI8.26	BD Biosciences
CD38	PE-CF594	HIT2	BD Biosciences
CD4	BV605	RPA-T4	BD Biosciences
CD40	BV510	5C3	BD Biosciences
CD45RO	BV570	UCHL1	Biolegend
CD71	PE	CY1G4	Biolegend
CD72	BV711	J4-117	BD Biosciences
CD86	PE-Cy5	IT2.2	Biolegend
CD95	PerCP-Cy5.5	DX2	BD Biosciences
CXCR3	AF700	1C6/CXCR3	BD Biosciences
CXCR5	AF488	RF8B2	BD Biosciences
FcRL5	PE	509F6	BD Biosciences
ICOS	PerCP-eFluor 710	ISA-3	Invitrogen
IgD	BV421	IA6-2	BD Biosciences
IL-21R	BV786	17A12	BD Biosciences
IL-6R	BB515	M5	BD Biosciences
IRF4	eFluor 450	3E 4	Invitrogen
IRF8	PerCP-eFluor 710	V3GYWCH	Invitrogen
Ki67	BV480	B56	BD Biosciences
PD-1	BV650	EH12.1	BD Biosciences
T-bet	PE	4B10	BD Biosciences
